# Higher expression of the strawberry xyloglucan endotransglucosylase/hydrolase genes *FvXTH9* and *FvXTH6* accelerates fruit ripening

**DOI:** 10.1111/tpj.14512

**Published:** 2019-10-08

**Authors:** Lucia D. Witasari, Fong‐Chin Huang, Thomas Hoffmann, Wilfried Rozhon, Stephen C. Fry, Wilfried Schwab

**Affiliations:** ^1^ Biotechnology of Natural Products Technische Universität München Liesel‐Beckmann‐Str. 1 85354 Freising Germany; ^2^ Department of Food and Agricultural Product Technology Faculty of Agricultural Technology Universitas Gadjah Mada Jl. Flora No. 1 – Bulaksumur Yogyakarta Indonesia; ^3^ Biotechnology of Horticultural Crops TUM School of Life Sciences Weihenstephan Technische Universität München Liesel‐Beckmann‐Str. 1 85354 Freising Germany; ^4^ Edinburgh Cell Wall Group Institute of Molecular Plant Sciences The University of Edinburgh Daniel Rutherford Building The King's Buildings Edinburgh EH9 3BF UK

**Keywords:** *Fragaria vesca*, *Fragaria × ananassa*, FvXTH9, FvXTH6, xyloglucan, xyloglucan endotransglucosylase (XET), mixed‐linkage glucan:xyloglucan endotransglucosylase (MXE), cellulose:xyloglucan endotransglucosylase (CXE), localization assay, overexpression

## Abstract

Fruit softening in *Fragaria* (strawberry) is proposed to be associated with the modification of cell wall components such as xyloglucan by the action of cell wall‐modifying enzymes. This study focuses on the *in vitro* and *in vivo* characterization of two recombinant xyloglucan endotransglucosylase/hydrolases (XTHs) from *Fragaria vesca*, FvXTH9 and FvXTH6. Mining of the publicly available *F. vesca* genome sequence yielded 28 putative *XTH* genes. *FvXTH9* showed the highest expression level of all FvXTHs in a fruit transcriptome data set and was selected with the closely related *FvXTH6* for further analysis. To investigate their role in fruit ripening in more detail, the coding sequences of *FvXTH9* and *FvXTH6* were cloned into the vector pYES2 and expressed in *Saccharomyces cerevisiae*. FvXTH9 and FvXTH6 displayed xyloglucan endotransglucosylase (XET) activity towards various acceptor substrates using xyloglucan as the donor substrate. Interestingly, FvXTH9 showed activity of mixed‐linkage glucan:xyloglucan endotransglucosylase (MXE) and cellulose:xyloglucan endotransglucosylase (CXE). The optimum pH of both FvXTH9 and FvXTH6 was 6.5. The prediction of subcellular localization suggested localization to the secretory pathway, which was confirmed by localization studies in *Nicotiana tabacum*. Overexpression showed that *Fragaria × ananassa* fruits infiltrated with *FvXTH9* and *FvXTH6* ripened faster and showed decreased firmness compared with the empty vector control pBI121. Thus FvXTH9 and also FvXTH6 might promote strawberry fruit ripening by the modification of cell wall components.

## Significance Statement

This study suggests that xyloglucan endotransglucosylase/hydrolases from *Fragaria vesca*, namely FvXTH9 and FvXTH6, promote strawberry fruit ripening through involvement in the modification of cell wall components. Overexpression of *FvXTH9* and *FvXTH6* in *Fragaria × ananassa* fruits resulted in accelerated fruit softening.

## Introduction

Plant cells are surrounded by a primary cell wall that consists of polysaccharides, proteins and sometimes also lignin, and exhibits variability in composition and organization. The ripening‐associated softening of fleshy fruit such as *Fragaria* (strawberry) is related to the selective modification of cell wall architecture (Prasanna *et al*., 2007; Fry, 2017a). During fruit ripening, modifications in the cell wall structure are characterized by the solubilization of pectic polysaccharides and by a decrease in the polymer size of xyloglucan (Hayashi and Kaida, 2011; Paniagua *et al*., 2017). Furthermore, the alteration of linkages between the polymers, such as in the cellulose–hemicellulose interaction, in parallel with decreasing fruit firmness takes place (Brummell, 2006; Vicente *et al*., 2007).

Strawberry fruit have a short post‐harvest shelf life as a result of the dramatic reduction in firmness during ripening. Recent findings showed that strawberry softening is closely related to pectin metabolism (Paniagua *et al*., 2017). The middle lamella of the cortical parenchyma cells is extensively degraded throughout ripening in strawberry (Perkins‐Veazie, 1995). Moreover, the silencing of genes encoding enzymes acting on pectins in strawberry, such as pectate lyase (Jiménez‐Bermúdez *et al*., 2002; Santiago‐Doménech *et al*., 2008; Youssef *et al*., 2009), rhamnogalacturonan lyase (Molina‐hidalgo *et al*., 2013), endo‐polygalacturonase (Quesada *et al*., 2009; Posé *et al*., 2013) and β‐galactosidase (Paniagua *et al*., 2016), involved in polyuronide solubilization and pectin depolymerization, significantly enhanced fruit firmness at ripening.

Xyloglucan, a highly significant hemicellulose, plays a crucial role in the determination of the physical properties of the cell wall during growth (Albersheim *et al*., 2011; Park and Cosgrove, 2015; Fry, 2017b). Xyloglucan possesses a 1,4‐β‐glucan backbone with 1,6‐α‐xylosyl residues along the backbone. In dicotyledons, some of the xylose residues are β‐d‐galactopyranosylated at *O*‐2 and some of the galactose residues are α‐l‐fucopyranosylated at *O*‐2. As the 1,4‐β‐glucan backbone can hydrogen‐bond to cellulose microfibrils, xyloglucan probably contributes to the inextensibility of the cell wall when it tethers adjacent microfibrils and to the loosening of the cell wall when it is degraded (Fry, 1989; Hayashi, 1989).

Several studies have analysed hemicellulose depolymerization during strawberry ripening (Huber, 1984;  Nogata *et al*., 1996;  Rosli *et al*., 2004). It has been suggested that endo‐β‐1,4‐glucanases modify the cellulose–xyloglucan network. Nevertheless, the downregulation of endo‐β‐1,4‐glucanase in *Fragaria × ananassa* Duch did not alter fruit firmness (Wooley *et al*., 2001; Palomer *et al*., 2006; Mercado *et al*., 2010).

The discovery of the enzyme XTH (xyloglucan endotransglucosylase/hydrolase) in the early 1990s (Baydoun and Fry, 1989; Smith and Fry, 1991; Fry *et al*., 1992; Nishitani and Tominaga, 1992) has provided a candidate that is considered a factor in cell wall modification leading to fruit softening. XTHs are the best‐known examples of transglycanases, a class of enzymes that catalyse polysaccharide:polysaccharide transglycosylation reactions involving substrates such as xyloglucan, mixed‐linkage (1→3, 1→4)‐β‐d‐glucan (MLG), cellulose, xylans and mannans (Franková and Fry, 2013). XTHs can catalyse the endolytic cleavage of xyloglucan polymers and the re‐joining of the newly generated reducing ends to other xyloglucan molecules, which is referred to as xyloglucan endotransglucosylase (XET) activity. In addition, XTHs can also show xyloglucan endohydrolase (XEH) activity, where water is used as an acceptor, and thus the xyloglucan molecule is hydrolysed (Fry *et al*., 1992; Nishitani and Tominaga, 1992; Thompson and Fry, 2001; Rose *et al*., 2002; Shi *et al*., 2015).

**Figure 1:**
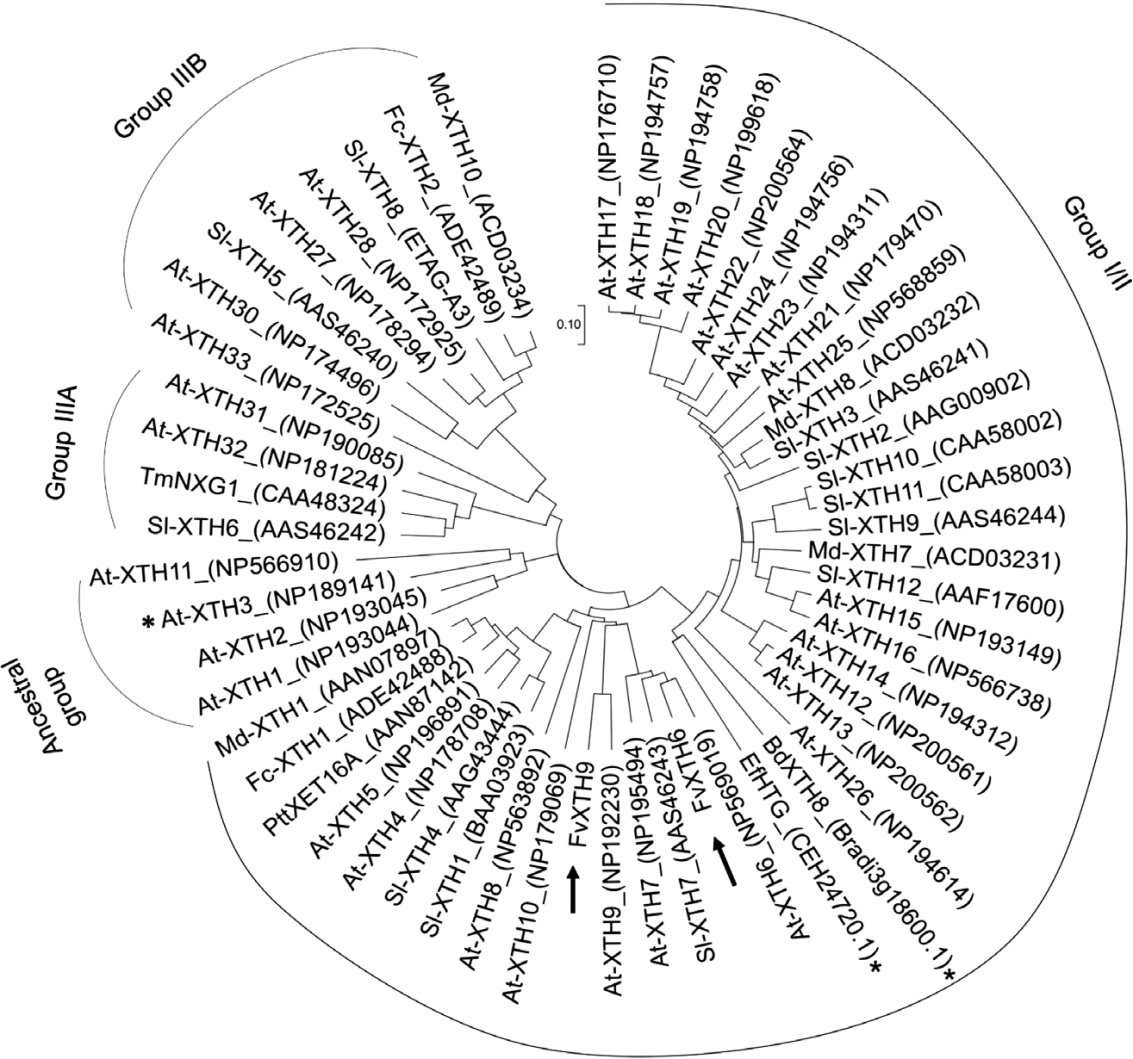
Phylogenetic tree of xyloglucan endotransglucosylase/hydrolases (XTHs) from different species. FvXTH9 and FvXTH6 protein sequences (indicated by arrows) were aligned with XTHs from *Arabidopsis thaliana* (At), *Brachypodium distachyon* (Bd), *Equisetum fluviatile* (Ef), *Fragaria chiloensis* (Fc), *Fragaria vesca* (Fv), *Malus *×* domestica* (Md), *Populus tremula *×* Populus tremuloides* (Ptt), *Solanum lycopersicum* (Sl) and *Tropaeolum majus* (Tm). EfHTG (hetero‐*trans*‐β‐glucanase) and BdXTH8 possess mixed‐linkage glucan:xyloglucan endotransglucosylase (MXE) activity, whereas AtXTH3 shows cellulose:xyloglucan endotransglucosylase (CXE) activity (indicated by asteriks). The phylogenetic tree was constructed by the neighbour‐joining method with 5000 bootstrap replications using mega 7. The GenBank accession numbers are indicated in the figure. Groups I/II, IIIA and IIIB show the different groups of XTHs.

The XTHs are classified together with the lichenases, which hydrolyse MLGs, in glycoside hydrolase family 16 (GH16, http://www.cazy.org/GH16.html; Planas, 2000; Hrmova *et al*., 2007; Lombard *et al*., 2014; Behar *et al*., 2018). The sequence similarity between XTHs and lichenases suggests that XTHs might be able to use MLG as an alternative donor substrate (Strohmeier *et al*., 2004). Indeed, it has been shown that an *Equisetum* hetero‐*trans*‐β‐glucanase (EfHTG; Figure 1) that is closely related to XTHs can catalyse transglucosylation reactions with MLG or cellulose (in preference to xyloglucan) as donor substrate and xyloglucan oligosaccharides (XGOs) as acceptor substrate (Fry *et al*., 2008; Simmons *et al*., 2015). The corresponding activities are termed MXE (MLG:xyloglucan endotransglucosylase) and CXE (cellulose:xyloglucan endotransglucosylase). Shinohara *et al*. (2017) recently showed that AtXTH3, a member of the ancestral group of XTHs (Figure 1) can catalyse cellulose:xyloglucan‐oligosaccharide (CXE) and cellulose:cellulose‐oligosaccharide transglycosylation, in addition to XET activity (xyloglucan:xyloglucan‐oligosaccharide transglycosylation).

The XTHs represent a huge multigene family, with 33 members in *Arabidopsis thaliana* (Yokoyama and Nishitani, 2000), 25 members in *Solanum lycopersicum* (tomato) (Saladié *et al*., 2006), 41 members in *Populus* (poplar) (Geisler‐Lee *et al*., 2006), 29 members in *Oryza sativa* (rice) (Yokoyama *et al*., 2004) and 22 members in *Hordeum vulgare* (barley) (Strohmeier *et al*., 2004). XTHs have also been characterized in several fruits, such as *Actinidia deliciosa* (kiwifruit) (Redgwell and Fry, 1993; Atkinson *et al*., 2009), *Malus domestica* (apple) (Atkinson *et al*., 2009) and *Diospyros* (persimmon) (Han *et al*., 2016). Recently, only two divergent XTH genes, namely *Fc‐XTH1* and *Fc‐XTH2*, have been identified in strawberry (*Fragaria chiloensis*), but biochemical assays for their predicted XET and/or XEH activity have not yet been performed (Opazo *et al*., 2010). A recent study showed the identification of 26 putative XTH‐encoding genes, named as *FvXTH*s, and their transcriptomic analysis, but the corresponding proteins have not yet been further characterized (Opazo *et al*., 2017).

To determine the contribution of xyloglucan modification to strawberry fruit softening during ripening, we searched the genome sequence of the diploid strawberry *Fragaria vesca* ssp. *vesca* accession Hawaii 4 for putative *XTH* genes (Shulaev *et al*., 2011). Based on transcriptome data (Härtl *et al*., 2017) and quantitative polymerase chain reaction (qPCR) analysis, *FvXTH9* and *FvXTH6* were selected for further analyses. We compared the *in vitro* enzymatic characteristics, including donor/acceptor substrate preference and enzyme kinetics, of FvXTH6 and FvXTH9 heterologously produced in the yeast *Saccharomyces cerevisiae*, and performed *in vivo* localization assays in *Nicotiana tabacum* (tobacco) leaves and transient expression in *F. × ananassa* fruit. The results show that the metabolism of xyloglucan at the early stages of strawberry fruit development contributes to softening and promotes ripening.

## Results

### Selection of candidate genes

Putative *XTH* genes were searched in the *F. vesca* ssp. vesca accession Hawaii 4 genome sequence to functionally characterize strawberry xyloglucan modifying enzymes in *Fragaria*. The transcript levels of the putative *XTH*s were analysed in a transcriptomic data set obtained from fruit (receptacle) of different developmental stages (green, white and ripe) of three *F. vesca* varieties (Reine de Vallées, Hawaii 4 and Yellow Wonder; Härtl *et al*., 2017; Figure S1). *FvXTH9* showed the highest expression level in green receptacles of all three genotypes. The *FvXTH9* transcript level in receptacle decreased with progressing ripening and was low in achenes throughout strawberry fruit development. A sequence analysis of different putative *F. vesca XTH* genes revealed that *FvXTH9* was closely related to *FvXTH6* (48.5% amino acid sequence identity) (Figure S2). Therefore, *FvXTH9* and *FvXTH6* might be involved in strawberry fruit ripening and were selected for further investigation.

To confirm the transcriptome data, the expression patterns of *FvXTH9* and *FvXTH6* were examined by quantitative real‐time PCR in *F. vesca* fruit at different ripening stages as well as in leaf and flower tissues (Figure S3). *FvXTH9* was highly expressed in fully developed green fruit, whereas its expression level dropped in later stages. A high expression level of *FvXTH9* was also observed in the flower, whereas its mRNA abundance was very low in all other tissues investigated. The expression pattern of *FvXTH6* during fruit development resembled that of *FvXTH9*, with the highest level found in fully developed green fruit and with a clear decline in later stages. The absolute expression level of *FvXTH6* in fruit was much lower than that of *FvXTH9*. Similar to *FvXTH9*,* FvXTH6* was also highly expressed in flowers; however, *FvXTH6* was expressed at a relatively high level in young leaf tissue but at a low level in old leaf tissue. These data indicate that *FvXTH9* and *FvXTH6* might play an important role in fruit development and probably also in the flower. In addition, *FvXTH6* might be involved in cell wall modification in the young leaf.

### Phylogenetic analysis and amino acid sequence analysis of FvXTH9 and FvXTH6

The deduced protein sequence of FvXTH9 is 294 amino acids long, with a predicted molecular mass of 33.2 kDa and a pI of 5.46. FvXTH6 consists of 293 amino acids with a theoretical molecular mass and pI value of 33.3 kDa and 6.44, respectively. In contrast to the *F. vesca* genome database (Shulaev *et al*., 2011), FvXTH9 and FvXTH6 were recently named as FvXTH6 and FvXTH3, respectively (Opazo *et al*., 2017); however, we decided to keep the name of these two proteins as FvXTH9 and FvXTH6 according to the *F. vesca* annotation release 101. A phylogenetic tree of XTHs shows that FvXTH9 and FvXTH6 are closely related to At‐XTH9 and At‐XTH6, respectively (Figure 1). Based on the phylogenetic tree of XTHs from different species, FvXTH9 and FvXTH6 are classified in group I/II (Figure 1), together with the well‐characterized PttXET16A, a strict XET enzyme (Johansson *et al*., 2004). All members of group I/II studied to date, for example Arabidopsis XTH12, 13, 14, 17, 18, 19 and 26 (Maris *et al*., 2009, 2011), have proved to possess XET activity but not XEH activity. Within group III, the bryophyte‐free group, are subclades IIIA, containing AtXTH31 (Zhu *et al*., 2012; Kaewthai *et al*., 2013) and TmNXG1 (Baumann *et al*., 2007), with XEH activity, and IIIB, containing numerous XTHs such as AtXTH27 (Campbell and Braam, 1999), SlXTH5 and SlXTH8 (Miedes and Lorences, 2009), with only XET activity.

Additionally, a multiple alignment was generated to access relationships among group I/II of XTHs (Figure S4). FvXTH9 and FvXTH6 contain the conserved motif of glycoside hydrolase family 16, (D/N)E(I/L/F)DFEFLGN, which comprises the active‐site motif (Campbell and Braam, 1998; Planas, 2000; Johansson *et al*., 2004; Baumann *et al*., 2007), and also a potential N‐linked glycosylation site, N‐X‐S/T, indicating that these proteins possess the common structural features of XTHs (Figure S4). FvXTH9 contains the NEFDFEFLGN sequence at position 97–105, whereas FvXTH6 has the DELDFEFLGN sequence at position 107–115. FvXTH9 and FvXTH6 have N‐T‐T and N‐R‐T as putative N‐glycosylation motifs directly after the catalytic motif, respectively. Most XTH genes have the N‐glycosylation motif immediately or spaced by 5–15 amino acids from the catalytic motif (Kallas *et al*., 2005). These findings identify FvXTH9 and FvXTH6 as typical class‐I/II XTHs.

### XET activity and pH dependency

To study the potential differential roles of the two putative xyloglucan endotransglucosylase/hydrolases, the full‐length coding sequences of *FvXTH9* and *FvXTH6* were amplified from *F. vesca* Hawaii 4 cDNA and cloned into pYES2. Both FvXTH9 and FvXTH6 were expressed in the heterologous *S. cerevisiae* expression system (Figure S5). Radioactive XET activity assays were performed at various pH values using the radiolabelled reduced xyloglucan heptasaccharide XXXGol as acceptor substrate and tamarind xyloglucan as donor substrate. The pH optimum of XET activity of both FvXTH9 and FvXTH6 crude extract was 6.5 in 100 mm sodium succinate buffer (Figure S6). FvXTH9 and FvXTH6 were inactive in XET assays at a pH of 4.0 and below. Whereas FvXTH6 exhibited only 25% activity at pH 4.8, FvXTH9 still showed approximately 70% of its maximal activity at that pH level, indicating a higher acid tolerance. Both enzymes displayed 50% of their maximal activities at pH 7.0, which dropped below 30% at pH 8.0. Their activities in sodium phosphate buffer were lower than in sodium succinate buffer at the same pH level.

### Substrate specificity of FvXTH9 and FvXTH6 for donor and acceptor substrates indicates XET and MXE activities

**Figure 2:**
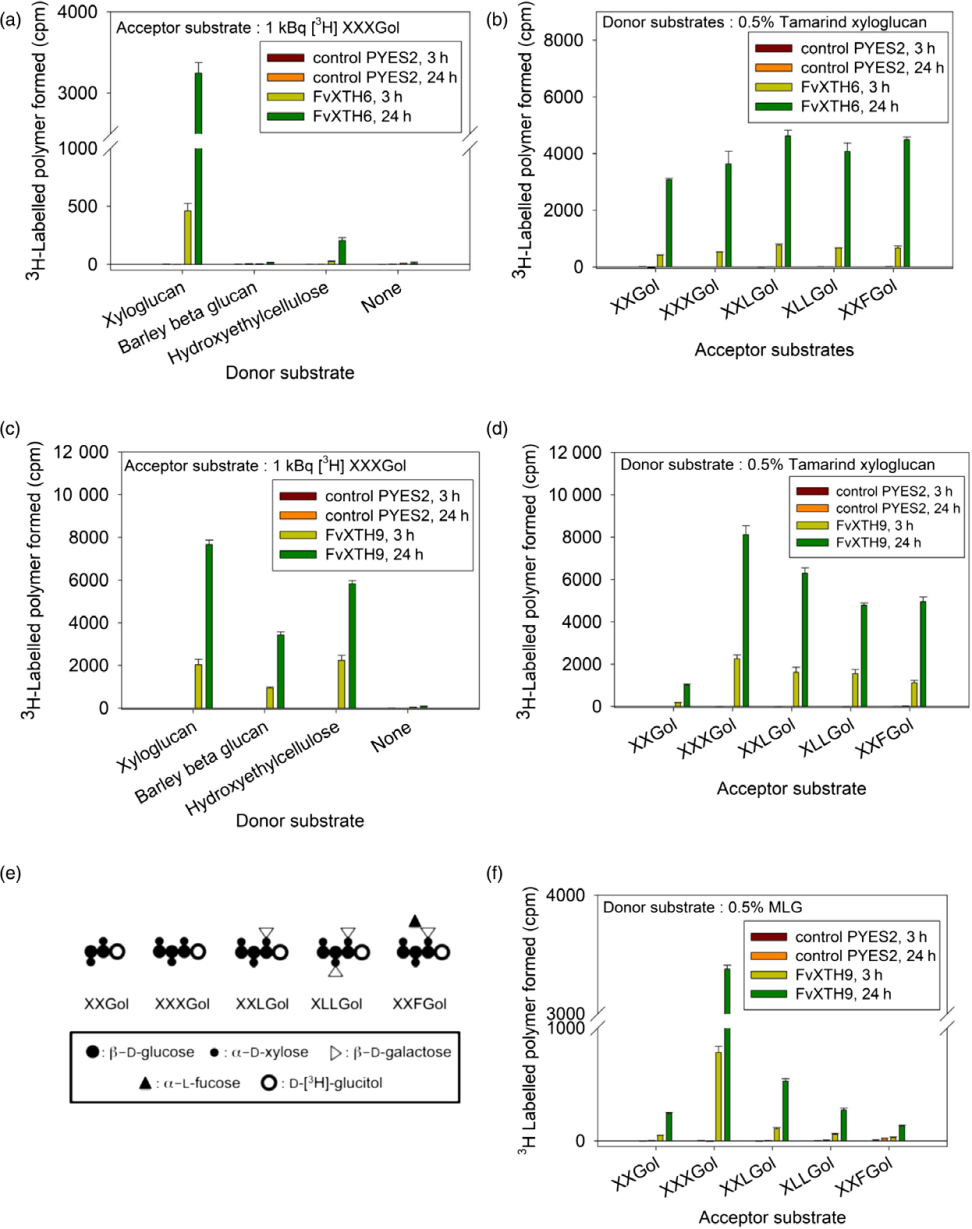
Xyloglucan endotransglucosylase (XET) assay of recombinant FvXTH6 (a, b) and FvXTH9 (c, d, f). Donor substrate preference for the XET assay tested with XXXGol as the acceptor substrate (a, c). Acceptor substrate preference for the XET assay tested with tamarind xyloglucan as donor substrate (b, d). Acceptor substrate preference for the mixed‐linkage glucan:xyloglucan endotransglucosylase (MXE) assay tested with mixed‐linkage (1→3, 1→4)‐β‐d‐glucan (MLG) as donor substrate (f). No XET activity was detected in crude protein extracts of empty vector‐transformed *Saccharomyces cerevisiae* cultures (control PYES2). Each assay was performed in four replicates; error bars show standard deviations. Unsubstituted glucose (Glc) residues are abbreviated as G, whereas X, L and F indicate Glc residues that are 6‐*O*‐substituted with α‐d‐Xylp, β‐d‐Galp‐(1‐2)‐α‐d‐Xylp and α‐l‐Fucp‐(1‐2)‐β‐d‐Galp‐(1‐2)‐α‐d‐Xylp side chains, respectively (e) (Fry *et al*., 1992).

Various acceptor substrates, including radiolabelled XXXGol, XXLGol, XLLGol, XXFGol and XXGol, and various donor substrates, such as tamarind xyloglucan, barley mixed‐linkage β‐glucan (MLG) and hydroxyethylcellulose (HEC), were used to determine the substrate specificity of the recombinant enzymes (Figure 2). FvXTH9 acted efficiently on all three donor substrates tested, with tamarind xyloglucan being the preferred one followed by HEC and barley MLG (Figure 2c). FvXTH9 therefore displayed XET and also MXE activity. The acceptor substrate preference for XET was in the following order: XXXGol > XXLGol > XLLGol ≈ XXFGol > XXGol (Figure 2d). For the MXE activity assay, it was in the following order: XXXGol > XXLGol > XLLGol > XXGol > XXFGol (Figure 2f). FvXTH6 used tamarind xyloglucan efficiently as a donor substrate, whereas only slight activity was observed for HEC and no activity was observed for MLG (Figure 2a). Acceptor substrates preference for FvXTH6 was XXLGol ≈ XXFGol > XLLGol > XXXGol > XXGol, although they showed only small differences in activity (Figure 2b).

### CXE activity

**Figure 3:**
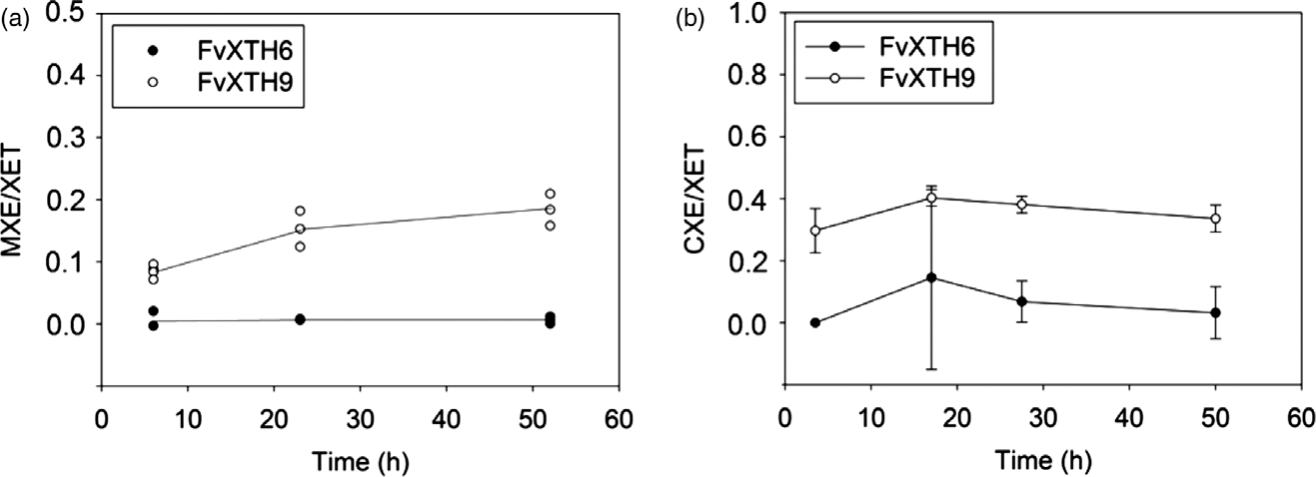
Mixed‐linkage glucan:xyloglucan endotransglucosylase (MXE)/xyloglucan endotransglucosylase (XET) and cellulose:xyloglucan endotransglucosylase (CXE)/XET activities of FvXTH9 and FvXTH6. MXE, CXE and XET assays used mixed‐linkage (1→3, 1→4)‐β‐d‐glucan (MLG), cellulose II (i.e. NaOH‐treated filter paper) and xyloglucan as donor substrates, respectively. All assays used radiolabelled XXXGol as the acceptor substrate. The results show the ratios of radioactivity in cpm after correction for zero‐donor controls. For MXE/XET activity (a), all values are the means of two assays. For CXE/XET activity (b), all values are the means of four assays, ±SE.

Finding MXE activity in a strawberry XTH was unexpected as dicots lack the donor substrate, MLG. The *Equisetum* enzyme responsible for MXE activity (Fry *et al*., 2008) was also found to possess CXE activity (Simmons *et al*., 2015), the donor substrate cellulose of which occurs in all land plants. Therefore, we tested the two strawberry XTHs for MXE activity (on MLG) compared with CXE activity (on cellulose II, i.e. NaOH‐treated filter paper) as the donor substrate. Both assays used radiolabelled XXXGol as the acceptor substrate. The result confirmed that FvXTH9 has MXE activity and revealed that it also possesses surprisingly high CXE activity. The MXE and CXE activities of FvXTH9 were 15–20% (Figure 3a) and 30–40% (Figure 3b) of the XET activity, respectively. FvXTH6 was confirmed to lack MXE activity; it also exhibited no appreciable CXE activity.

### Kinetic properties of FvXTH9 and FvXTH6

**Figure 4:**
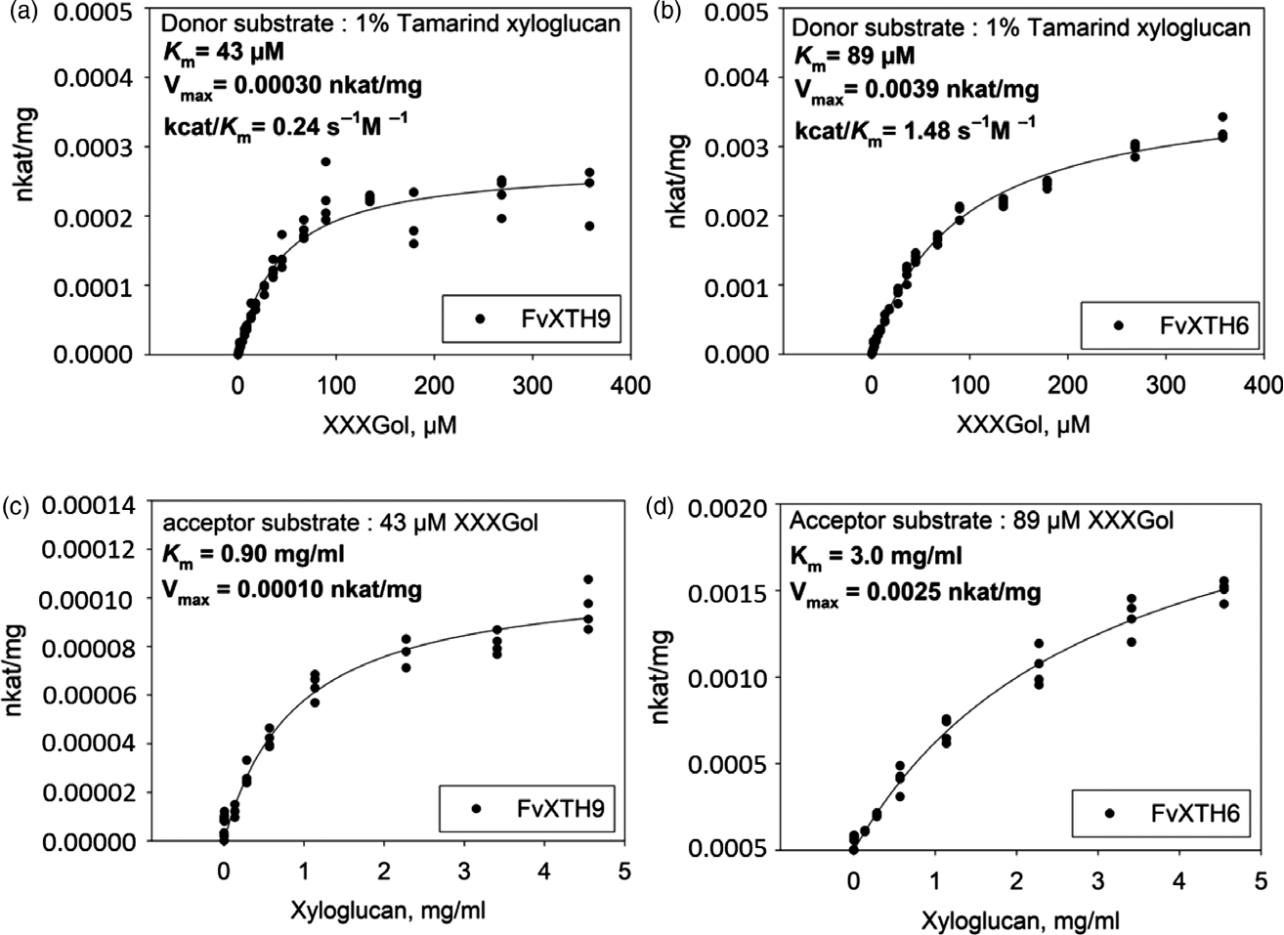
Effect of acceptor‐substrate concentration and donor‐substrate concentration on transglucosylation rate catalysed by 1.01 mg ml^−1^ FvXTH9 protein (a, c) and 0.86 mg ml^−1^ FvXTH6 protein (b, d). Each assay was performed in four replicates.

To investigate the kinetic parameters of FvXTH9 and FvXTH6 in more detail, both enzymes were expressed in yeast and purified as His_6_‐tagged protein (Figure S5) and assayed for XET activity. For XXXGol, FvXTH9 displayed a Michaelis constant (*K*_m_ of 43 μm and a maximal rate of reaction (*V*_max_ of 0.00030 nkat mg^−1^. Recombinant FvXTH6 showed a *K*_m_ of 89 μm and a *V*_max_ of 0.0039 nkat mg^−1^ for XXXGol. For xyloglucan, FvXTH9 showed a *K*_m_ of 0.90 mg ml^−1^ and a *V*_max_ of 0.00010 nkat mg^−1^. The *K*_M_ and *V*_max_ of FvXTH6 for xyloglucan were 3.0 mg ml^−1^ and 0.0025 nkat mg^−1^, respectively (Figure 4). *K*_m_ values for the donor substrate are quoted in mg ml^−1^, not μm, because XTHs are able to use any segment of the polysaccharide chain equally, not just one site per molecule as is the case with the acceptor substrate (Rose *et al*., 2002).

### Subcellular localization

Prediction of the subcellular localization for both FvXTH9 and FvXTH6 pointed to the endoplasmic reticulum (ER) using Predotar 1.04, the secretory pathway using TargetP 1.1 and MultiLoc2, and the cell wall using Plant‐mPLoc (Table S1). These predictions suggest that FvXTH9 and FvXTH6 travel from the ER via vesicles to the Golgi apparatus and finally to the cell membrane to be released into the apoplast.

**Figure 5:**
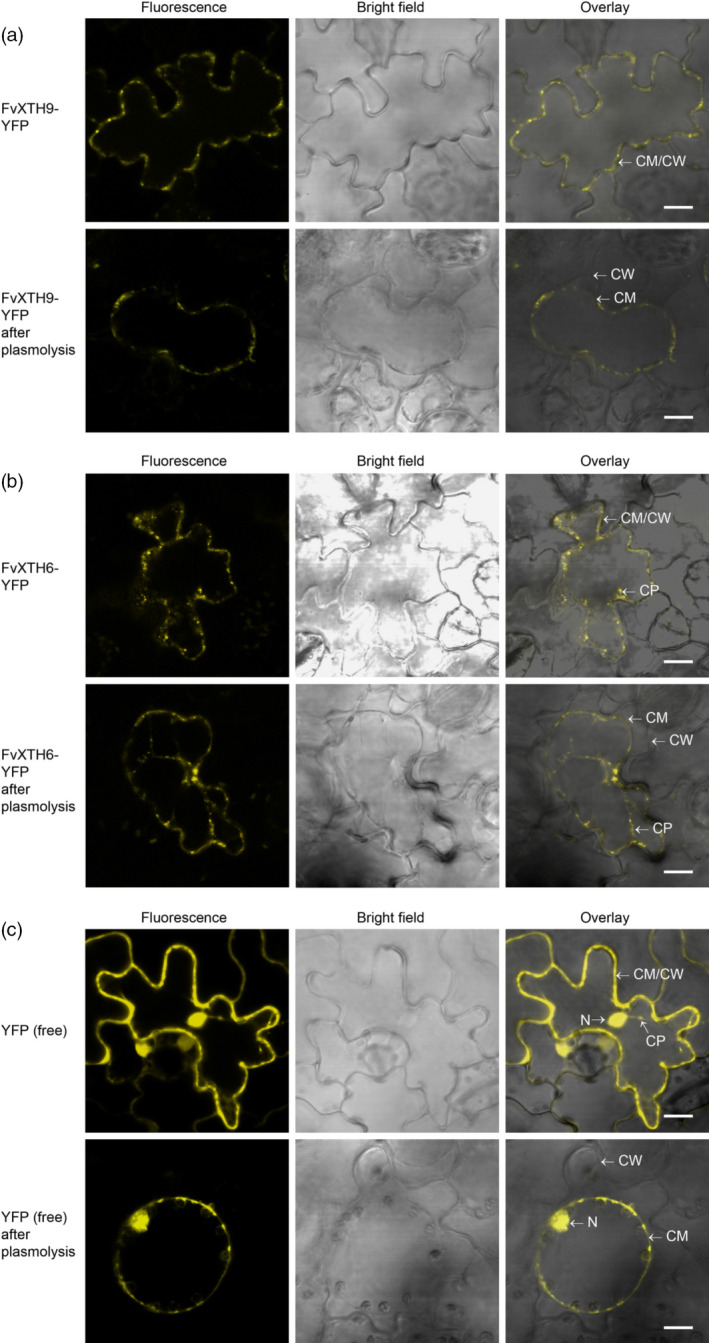
Localization of FvXTH9‐YFP (a), FvXTH6‐YFP (b), and control‐YFP (c) in *Nicotiana tabaccum* leaves. CW, cell wall; CM, cell membrane; CP, cytoplasm; N, nucleus. Scale bar: 10 μm.

In order to provide experimental evidence for the subcellular localization of FvXTH9 and FvXTH6 in plant cells, the full‐length coding sequence of *FvXTH9* or *FvXTH6* was fused with the *YFP* gene at the C terminal and placed under the control of the *35S* cauliflower mosaic virus (CaMV) promoter. The constructs were introduced into *Agrobacterium tumefaciens* GV3101/pSoup cells by transformation and infiltrated into *Nicotiana tabacum* leaves. As a control, a construct encoding free YFP was infiltrated in the same way. Confocal microscopy showed clear signals at the cell membrane and/or cell wall for both FvXTH9‐YFP (Figure 5a) and FvXTH6‐YFP (Figure 5b). For FvXTH6‐YFP, clear signals could also be observed in the cytoplasm. Interestingly, the signals of both proteins appeared mainly in spots of high intensity, indicating localization to subcellular structures, like vesicles, the ER or specific regions of the cell membrane. In contrast, the signal for free YFP was more evenly distributed, as is expected for a cytoplasmic protein (Figure 5c). To investigate a potential localization to the cell wall, infiltrated plant cells were treated with 0.5% (w/v) NaCl for 30 min to induce plasmolysis. Under these conditions no signals were visible in the cell wall whereas the spots in the cell were clearly visible, indicating that the proteins, if released from the cell, are not tightly associated with the cell wall. These results show that both FvXTH9 and FvXTH6 are localized to the cell membrane. FvXTH6 also appears in spots in the cytoplasm, which probably represent vesicles of the secretory pathway.

### Overexpression of XTH genes in strawberry fruit

**Figure 6:**
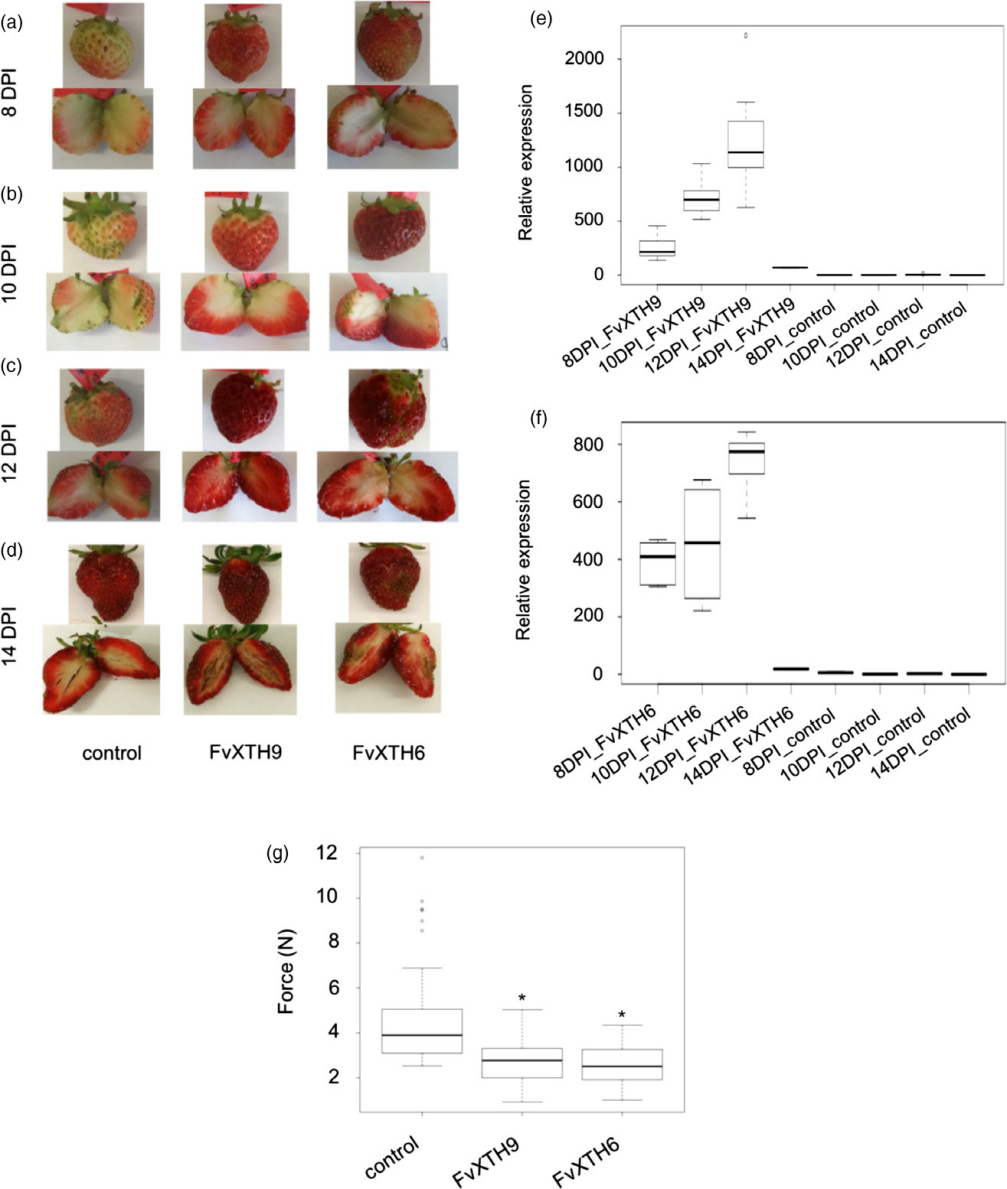
*Fragaria *×* ananassa* fruit phenotypes, qPCR analysis and texture analysis after agroinfiltration with *FvXTH9* and *FvXTH6*. Fruit phenotypes after (a) 8 days post infiltration (dpi), (b) 10 dpi, (c) 12 dpi and (d) 14 dpi. qPCR analysis of *FvXTH9* (e) and *FvXTH6* (f) in *F. *×* ananassa* after 8, 10, 12 and 14 dpi. qPCR data were obtained by analysing two or three biological replications and three technical replications. (g) Texture analyser was fitted with a 5‐mm flat probe. Each fruit (12 dpi) was penetrated to 5 mm at a speed of 0.5 mm sec^−1^ and the maximum force developed during the test was recorded in newtons (N). The data were obtained by analysing 60 fruits for each group. The asterisk indicates statistically significant differences (*P* < 0.05) between agroinfiltrated fruits with *XTH* s and the empty plasmid. Control fruit were infiltrated with *Agrobacterium tumefaciens* AgL0 carrying pBI121 empty plasmid.

In order to investigate the role of *FvXTH9* and *FvXTH6* in strawberry fruit ripening, both proteins were overexpressed in *F. × ananassa* fruit. As both genes showed decreasing transcripts during ripening (Figures S1 and S3), we decided to overexpress the genes rather than downregulate them in order to achieve maximum effects and thus clear results. White immature *F. × ananassa* fruits, while still attached to the plant, were evenly infiltrated with *A. tumefaciens* AGL0 containing the Ti vector pBI121 harbouring the full‐length coding sequence of *FvXTH9* or *FvXTH6*. As a control, fruits were infiltrated with agrobacteria possessing the empty vector pBI121. Infiltrated fruits were harvested 8, 10, 12 and 14 days post infiltration (dpi). Fruits infiltrated with *FvXTH9* and *FvXTH6* ripened faster (Figure 6a–d) than control fruits. Gene expression analysis indicated that *XTH* genes were highly expressed in both *FvXTH9* and *FvXTH6* infiltrated fruits compared with control pBI121 fruits (Figure 6e,f). Gene expression levels of *FvXTH9* and *FvXTH6* increased from 8 to 12 dpi.

### Effect on fruit firmness and metabolite levels

**Figure 7:**
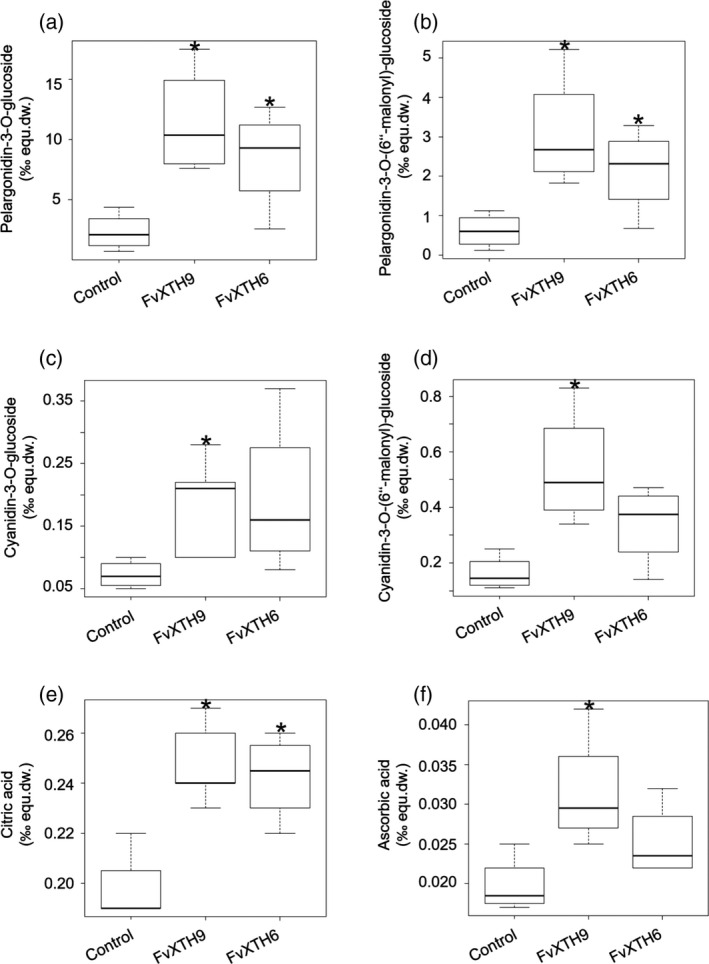
Metabolite analysis of *Fragaria × ananassa* fruit after agroinfiltration of *FvXTH9* and *FvXTH6*. Relative concentration of (a) pelargonidin‐3‐*O*‐glucoside, (b) pelargonidin‐3‐*O*‐(6′‐malonyl)‐glucoside, (c) cyanidin‐3‐*O*‐glucoside (d) cyanidin‐3‐*O*‐(6′‐malonyl)‐glucoside, (e) citric acid and (f) ascorbic acid. The data were calculated as ‰ equivalent internal standard of the dry weight (‰ equ.dw.) from between four and six fruits (10 dpi) for each sample. Control fruit were infiltrated with *Agrobacterium tumefaciens* AgL0 carrying pBI121 empty plasmid. The asterisk indicates statistically significant differences (*P* < 0.05) between agroinfiltrated fruits with *XTH* s and the empty plasmid.

To evaluate the effect of *FvXTH9* and *FvXTH6* upregulation on the rigidity of strawberry fruits, the firmness of the fruits was measured using a texture analyser. *FvXTH9* and *FvXTH6* overexpressing fruits were clearly softer than control pBI121 fruits, as significantly less force was required to penetrate the fruit tissue (Figure 6g). Metabolites were identified and quantified using LC‐MS analysis and the internal standard method. The results showed that the levels of ripening‐related anthocyanins, such as pelargonidin‐3‐*O*‐glucoside, pelargonidin‐3‐*O*‐(6′‐malonyl)‐glucoside, cyanidin‐3‐*O*‐glucoside and cyanidin‐3‐*O*‐(6′‐malonyl)‐glucoside were increased in both the *FvXTH9*‐ and *FvXTH6*‐overexpressed fruits (Figure 7a–d). The same result was found for citric acid and for ascorbic acid (Figure 7e,f); however, the concentrations of metabolites known to accumulate in immature fruits, such as the proanthocyanidins catechin, epicatechin and the dimer epiafzelechin‐epicatechin, remained unchanged, except for the epicatechin dimer, which showed low levels in *FvXTH9*‐overexpressed fruits (Figure S7). The other metabolites showed mixed results. Kaempferol derivatives strongly accumulated in transgenic fruits whereas concentrations of quercetin derivatives were not affected.

## Discussion

Xyloglucan endotransglucosylase/hydrolases (XTHs) are cell wall‐modifying enzymes that have been implicated in fruit softening during ripening. Here, we functionally characterized two phylogenetically related XTHs from *F. vesca*, which are differently transcribed during fruit development. *FvXTH9* and *FvXTH6* showed low transcript levels at the early maturation stage of *F. vesca* (small green stage), the highest levels at the large green stage and then a decrease at the end of ripening (ripe stage) (Figure S3). This pattern is comparable with *FcXTH1* (*F. chiloensis*), with high levels in large green (LG) and turning (T) stages (Opazo *et al*., 2010). In kiwifruit, the genes *AdXTH1* and *AdXTH4* have a ‘down–up–down’ expression profile during softening (Atkinson *et al*., 2009). In persimmon fruit, the expression of *DkXTH1*,* DkXTH4* and *DkXTH5* was very high in immature growing fruit and peaked before the mature stage (Han *et al*., 2015). Phylogenetic analysis revealed that FvXTH9 and FvXTH6 belong to group I/II. The classification of XTHs reflects the different biochemical mechanisms of enzyme action (Rose *et al*., 2002). Members of group I/II and IIIB predominantly exhibit XET activity alone (Nishitani and Tominaga, 1992; Rose *et al*., 2002; Maris *et al*., 2011), whereas members of group IIIA mainly exhibit XEH activity (Fanutti *et al*., 1993; Baumann *et al*., 2007; Zhu *et al*., 2012; Kaewthai *et al*., 2013). The XET activities of FvXTH9 and FvXTH6 showed optima at pH 6.5 (Figure S6). The fact that both enzymes have very similar pH–activity profiles is not surprising, as they are phylogenetically very closely related (Yokoyama and Nishitani, 2001; Baumann *et al*., 2007) and acidic pH optima are typical of XTHs (Purugganan *et al*., 1997; Campbell and Braam, 1999; Steele and Fry, 2000; Maris *et al*., 2011). Root hair formation has been established as a model to study the pH of the cell wall during localized growth in plants, which is associated with cell wall modification. In these studies it was shown that the first morphological changes were accompanied by pH changes of the initiation site from pH 5.0 to pH 4.5, and during tip growth to pH 6.0, whereas the cytoplasmic pH increased from pH 7.3 to pH 7.7 (Bibikova *et al*., 1998). Therefore, XTHs show the highest activity in the cell wall at the site of their substrates. Similarly, the root cap apoplast acidified from pH 5.5 to pH 4.5 within 2 min of gravistimulation, and the cytoplasmic pH increased from pH 7.2 to pH 7.6 (Fasano *et al*., 2001).

Xyloglucan is the preferred donor substrate for both FvXTH9 and FvXTH6. Crude extract of yeast expressing FvXTH9 also showed some enzyme activity using barley MLG as well as HEC as donors (Figure 2c). FvXTH6 could use HEC to some extent, whereas barley MLG was not tolerated as a donor substrate (Figure 2a), indicating that FvXTH6 has no MXE activity. XTHs attack not only the xyloglucan polymer but also some soluble artificial substrates. A pure XTH isoenzyme from barley (HvXET5) could react with xyloglucan (100% rate), HEC (44%), water soluble cellulose acetate (WSCA) (5%) and carboxymethylcellulose (CMC) (0.4%). HvXET5 had very low activity on MLG as a donor (0.2% rate), and real (insoluble) cellulose was not tested (Hrmova *et al*., 2007). For AtXTH12, 13, 17, 18, and 19 enzymes, the non‐xyloglucan polymers were preferred in the order WSCA > HEC > MLG > CMC (Maris *et al*., 2011). Enzyme extracts from young shoots of *Equisetum* and the grass *Holcus lanatus* share similar donor–substrate profiles, with relative activities on xyloglucan:WSCA:HEC:CMC being 100:(20–24):(11–17):(0.4–1.5) (Fry *et al*., 2008). In contrast to the predominant XET activity of *Equisetum*, caused by standard XTH proteins, the MXE activity of *Equisetum* was found to result from a unique hetero‐*trans*‐β‐glucanase (Fry *et al*., 2008; Simmons *et al*., 2015).

The acceptor substrate preference was studied on a selection of oligosaccharidyl [^3^H]alditols with various side chains and backbones differing in the number of glucose units. XET activity for FvXTH9 and FvXTH6 differ slightly in their oligosaccharide acceptor‐substrate preference. The best acceptor substrate for FvXTH9 is XXXGol, in agreement with the preference of the MXE‐active *Equisetum* enzyme, HTG (Simmons *et al*., 2015), followed by XXLGol, whereas both XXLGol and XXFGol are preferred acceptor substrates for FvXTH6, followed by XLLGol. FvXTH9 and FvXTH6 showed lower activity with the doubly galactosylated acceptor substrate (XLLGol) and also with the fucosylated substrate (XXFGol). The fucosyl residues are predicted to alter the conformation of the xyloglucan polymer (Levy *et al*., 1997). This altered conformation may reduce the binding of XTHs. The activity with the pentasaccharide XXGol compound is relatively low for both enzymes. Eight native XTH isoenzymes from *Vigna* and *Brassica* have the consistent order of acceptor‐substrate preference: XLLGol > XXLGol > XXXGol > XXGol (XGol was ineffective) (Steele and Fry, 2000). Acceptor preferences for XET activity were XXXGol > XXFGol > XXLGol > XLLGol > XLFGol for *A. thaliana* AtXTH31 (which has much higher XEH activity than XET activity) and XXXGol > XLLGol > XLFGol > XXLGol > XXFGol for AtXTH15 (which lacks XEH activity) (Shi *et al*., 2015).

Our results also showed that FvXTH9 (but not FvXTH6) has not only XET activity but also MXE activity (15–20% of XET) because barley β‐glucan functioned as a donor substrate. For MXE, FvXTH9 showed a strong preference for XXXGol as the acceptor substrate, whereas all other compounds tested were only inefficiently used (Figure 2f). The order of the acceptor preference of MXE activity is slightly different from that of XET activity, but the relative activities of the less preferred substrates are considerably lower. In the case of MXE activity of *Equisetum* (Fry *et al*., 2008; Simmons *et al*., 2015), the best acceptor substrate was XXXGol (with both XLLGol and XXGol being much less effective), whereas XLLGol was the best acceptor substrate for XET, followed by XXXGol and then XXGol.

The discovery that a strawberry transglucanase (FvXTH9) can use MLG as a donor substrate was unexpected, as MLG does not occur in dicots; however, FvXTH9 shows sequence similarity and phylogenetic relationship to EfHTG and BdXTH8, two characterised heterotransglycosylases (Figures 1 and S8). In *Equisetum*, the protein responsible for MXE activity (HTG) also possesses CXE activity. We therefore tested whether strawberry XTHs also have CXE activity, as the substrates in that case (cellulose + xyloglucan) are ubiquitous in land plants. Indeed, we found that FvXTH9 also mediates endotransglucosylation from cellulose to xyloglucan oligosaccharides (i.e. CXE activity). The CXE was 30–40% of the XET activity, measured at various time points. This is a very high relative CXE activity for an angiosperm transglucanase, comparable with the ~45% reported for AtXTH3 (Shinohara *et al*., 2017). It is highly plausible that FvXTH9 may catalyse cellulose–xyloglucan grafting in the ripening strawberry as a physiological reaction in addition to its better‐known xyloglucan–xyloglucan grafting. It is difficult to imagine how CXE action (cellulose–xyloglucan grafting) could contribute to fruit softening, however, as it might be envisaged to strengthen the cell walls (Simmons *et al*., 2015).

The XET catalytic properties of the purified recombinant FvXTH9 and FvXTH6 proteins towards tamarind xyloglucan and XXXGol were studied and produced classical hyperbolic Michaelis–Menten curves (Figure 4). FvXTH9 showed a higher affinity for XXXGol (*K*_m_ = 43 μm) than did FvXTH6 (*K*_m_ = 89 μm). These *K*_m_ values for XXXGol of both enzymes are within the range of *K*_m_ values with other XTHs. Arabidopsis XTH15 and XTH31 had *K*_m_ values of 31 and 86 μm, respectively (Shi *et al*., 2015), whereas XET from *Equisetum* and barley showed *K*_m_ values of 80 μm (Fry *et al*., 2008) and 40 μm (Hrmova *et al*., 2009), respectively. In contrast, the unique hetero‐transglycanase, *Equisetum* HTG, has an extremely high affinity for XXXGol (~0.5–1.0 μm; Simmons *et al*., 2015).

The calculation of the catalytic properties of FvXTH9 towards xyloglucan revealed a *K*_m_ of 0.90 mg ml^−1^, indicating a higher affinity than with FvXTH6 (*K*_m_ = 3.0 mg ml^−1^). These results were comparable with other XETs. XET activity *K*_m_ values for xyloglucan were reported in *Equisetum* (*K*_m_ = 0.35 mg ml^−1^; Fry *et al*., 2008), in kiwifruit (*K*_m_ = 0.6 mg ml^−1^; Schröder *et al*., 1998), in Arabidopsis (XTH22; *K*_m_ = 1.8 and 0.6 mg ml^−1^ for fucosylated and non‐fucosylated xyloglucans, respectively; Purugganan *et al*., 1997; and XTH15, *K*_m_ = 2.87 mg ml^−1^; Shi *et al*., 2015). There was no decrease in the rate of ^3^H incorporation at the highest concentrations of non‐radioactive xyloglucan, indicating that 4.5 mg ml^−1^ polysaccharide (≈5 μm) did not appreciably compete with [^3^H]XXXGol as the acceptor substrate.

The prediction of subcellular localization revealed localization to the secretory pathway, or to organelles associated with the secretory pathway, for both FvXTH9 and FvXTH6. This was confirmed by confocal microscopy of tobacco leaves agroinfiltrated with YFP‐tagged versions of FvXTH9 and FvXTH6, which localize to the vesicle of the secretory pathway and the cell membrane (Figure 5a,b). The full‐length AtXTH31‐GFP fusion protein was targeted to the plasma membrane by an N‐terminal signal peptide (Zhu *et al*., 2012). AtXTH33 was also localized to the plasma membrane (Ndamukong *et al*., 2009). As xyloglucan is synthesized in the Golgi and transported via exocytosis to undergo transglycosylation immediately upon release into the cell wall (Thompson and Fry, 2001), FvXTH9 and FvXTH6 may be well positioned for catalysing this process on newly secreted xyloglucan.

To confirm whether FvXTH9 and FvXTH6 are involved in fruit ripening and softening, overexpression of the target genes in strawberry fruit was performed. qPCR analysis indicated that *XTH* genes were successfully overexpressed in both *FvXTH6*‐ and *FvXTH9*‐agroinfiltrated fruits. In contrast, significantly lower transcript levels of *FvXTH9* and *FvXTH6* were detected in control pBI121 fruits. The expression patterns of both genes increased from 8 to 12 dpi and then decreased significantly by 14 dpi (Figure 6e,f).

Overexpression of gene products in plants may change the phenotypes. The results showed that fruits infiltrated with *FvXTH9* and *FvXTH6* exhibited accelerated colour change and ripened faster compared with the control pBI121 (Figure 6a–d). To support this finding, the differences in fruit firmness at 12 dpi were recorded. Compared with control pBI121 fruit, the *FvXTH9* and *FvXTH6* infiltrated fruits showed decreased firmness (Figure 6g). The texture analysis supports the observation that fruits of both transgenics ripened faster than the control fruit. Similarly, overexpression of *DkXTH8* in tomato fruit led to accelerated colour change and decreased firmness, compared with wild‐type fruit (Han *et al*., 2016), whereas UV‐C irradiation of tomato fruit reduced the activity of cell wall‐degrading enzymes and delayed the ripening of tomato fruit. Irradiated fruit were firmer than control fruit (Barka *et al*., 2000). Overexpression of AtXTH9 (which is closely related to FvXTH9) caused pronounced cell expansion and stem growth (Shin *et al*., 2006). The genes *FvXTH9* and *FvXTH6* might promote strawberry fruit softening through involvement in cell wall restructuring. Metabolite analysis supported the hypothesis of accelerated fruit ripening as a result of overexpression of *FvXTH9* and *FvXTH6*. The level of anthocyanins such as pelargonidin‐3‐*O*‐glucoside and pelargonidin‐3‐*O*‐(6′‐malonyl)‐glucoside was significantly higher in the infiltrated fruits (Figure 7a,b), similar to citric acid and ascorbic acid (Figure 7e,f). These metabolites are known to accumulate during strawberry fruit ripening (Griesser *et al*., 2008; Zhang *et al*., 2011; Ornelas‐Paz *et al*., 2013). Interestingly, kaempferol glucoside and glucuronide showed higher concentrations in transgenic fruits, whereas levels of quercetin derivatives remained unchanged. This confirmed the observation that the concentration of kaempferol glucoside increased significantly in the final stages of strawberry fruit ripening, whereas the quercetin derivative showed only a moderate increase (Griesser *et al*., 2008).

In conclusion, two functional xyloglucan endotransglucosylase/hydrolases, FvXTH9 and FvXTH6, were identified from *F. vesca. FvXTH9* is highly expressed in immature fruits and then the transcript levels decrease until full maturity. The recombinant FvXTH9 and FvXTH6 proteins showed XET activity. In addition, FvXTH9 also has MXE activity and CXE activity. Overexpression of FvXTH9 and FvXTH6 resulted in accelerated fruit softening in strawberry fruit. Thus, FvXTH9 and probably also FvXTH6 are likely to be capable of modifying the structure of xyloglucan in the cell wall.

## Experimental procedures

### Plant material and reagents

Octoploid strawberry plants (*F. × ananassa* cv. Elsanta) and diploid (*F. vesca* cv. Hawaii 4) were obtained from Kraege Beerenpflanzen (https://kraege.de). Strawberry plants were grown under glasshouse conditions (16‐h light/8‐h dark) in Dünast, Freising, Germany. *Nicotiana tabacum* cv. Samsum was grown at 25°C with a 16‐h photoperiod under artificial light at 120 μmol m^−2^ sec^−1^ irradiance, provided by Osram Fluora lamps (Osram, https://www.osram.com/cb). Chemicals and solvents were obtained from Sigma‐Aldrich (https://www.sigmaaldrich.com), Carl Roth (https://www.carlroth.com), VWR International (https://www.vwr.com) and J.T. Baker (now part of Avantor, https://www.avantorsciences.com), unless otherwise noted. Tamarind seed xyloglucan was a generous gift from Dr K. Yamatoya, Sumitomo Dainippon Pharma Co., Ltd. (https://www.ds-pharma.com). Non‐radioactive XXXGol and medium‐viscosity barley MLG were obtained from Megazyme (https://www.megazyme.com). Tritiated XXXGol (specific activity 720 MBq μmol^−1^), XXGol (19 MBq μmol^−1^), XLLGol (53 MBq μmol^−1^), XXLGol (53 MBq μmol^−1^) and XXFGol (27 MBq μmol^−1^), prepared by NaB^3^H_4_ reduction of the corresponding reducing oligosaccharides (Hetherington and Fry, 1993), were obtained from EDIPOS ( http://fry.bio.ed.ac.uk//edipos.html).

### Gene expression analysis

Total RNA was extracted from *F. vesca* Hawaii 4 fruit (Liao *et al*., 2004). First‐strand cDNA was synthesized by reverse transcription with 1 μg of total RNA, random hexamer primer and M‐MLV reverse transcriptase (Promega, https://www.promega.com). RT‐PCR was performed in a StepOnePlus^™^ real‐time PCR system (Applied Biosystems, now ThermoFisher Scientific, https://www.thermofisher.com). The qRT‐PCR data for FvXTHs were normalized against the expression levels of the interspacer *26S*–*18S* RNA housekeeping gene. Gene‐specific primers and interspacer (IS) primers were used to amplify the target gene and the IS gene, respectively (Table S2). The RT‐PCR mixture was 10 μl of 2× Power SYBR Green Mix, 0.8 μl of forward primer (10 μm), 0.8 μl of reverse primer (10 μm), 2 μl of cDNA and 6.8 μl of ultrapure H_2_O. The dilutions of cDNA for target gene and IS gene were 20× and 8000×, respectively. The absence of unspecific amplicon was confirmed by melting curve profiles. Three technical and two or three biological replicates of each sample were performed. The results were calculated using the ΔΔ*C*_t_ method.

### Cloning and expression

*FvXTH9* (882 bp) and *FvXTH6* (880 bp) were amplified from *F. vesca* cDNA using specific primers. For *FvXTH6*, the forward primer was FP_FvXTH6_PYES2 and the reverse primer was RP_FvXTH6_PYES2. For *FvXTH9*, the forward and reverse primers were FP_FvXTH9_PYES2 and RP_FvXTH9HIS_PYES2, respectively (Table S2). Denaturation was carried out at 95°C for 45 sec, annealing at 55°C for 30 sec and elongation at 72°C for 1 min, in 35 cycles. Cloning was conducted using the pYES2 vector. Transformation was performed in two steps, first using *Escherichia coli* NEβ 10 cells for maintaining recombinant plasmid and subsequently *S. cerevisiae* INVSc1 cells for protein expression. The constructs obtained were sequenced to confirm the absence of error introduced by cDNA synthesis and/or PCR. Galactose induction was used to induce the expression of the protein of interest from the GAL1 promoter. A single colony of INVSc1 containing the pYES2 construct was inoculated into 15 ml of SC‐U medium consisting of 0.67% yeast nitrogen base (without amino acids), 2% carbon source [d‐glucose, 0.01% (adenine, arginine, cysteine, leucine, lysine, threonine, tryptophan, uracil], 0.005% (aspartic acid, histidine, isoleucine, methionine, phenylalanine, proline, serine, tyrosine, valine) and incubated overnight at 30°C at 2000 ***g***. Next day, the OD_600_ of the overnight culture was determined and diluted to obtain an OD_600_ of 0.4 in 50 ml of induction medium (SC‐U medium containing 2% galactose). The cells were pelleted by centrifugation at 1500 ***g*** for 5 min at 4°C and then inoculated into 50 ml of induction medium. The cells were grown at 30°C, 2000 ***g***. Cells were pelleted by centrifugation at 1500 ***g*** for 5 min at 4°C. Extracts from *S*. *cerevisiae* cells were prepared using breaking buffer (50 mm sodium phosphate, pH 7.4), 1 mm ethylenediaminetetraacetic acid (EDTA), 5% glycerol and 1 mm phenylmethylsulfonyl fluoride (PMSF), following the general protocol for small‐scale preparation of cell lysates using acid‐washed glass beads (Invitrogen, now ThermoFisher Scientific). Purification was carried out using a HisTrap FF column 5 ml (Invitrogen, now ThermoFisher Scientific) in a fast protein liquid chromatography (FPLC) system (ÄKTA system; GE Healthcare, https://www.gehealthcare.com). The column was equilibrated in his‐tag wash/bind buffer (50 mm sodium phosphate, pH 7.4, 0.3 m NaCl and 30 mm imidazole) and delivered at a flow rate of 0.5 ml min^−1^ for 30 min. The elution of target protein was performed using an isocratic gradient elution of 1× His elution buffer (50 mm NaPi, pH 7.4, 0.3 m NaCl, 250 mm imidazole) for 20 min with a flow rate of 0.5 ml min^−1^. Fractions (2 ml) were collected for enzyme assays.

### Sequence alignment and phylogenetic tree

Full‐length amino acid sequences of XTHs were obtained from the genome sequence of *F. vesca *ssp. vesca accession Hawaii 4 (Shulaev *et al*., 2011). The phylogenetic tree was constructed by the neighbour‐joining method with 5000 bootstrap replications using mega 7. The amino acid sequence alignment of group‐I XTHs was compiled using alignx vector nti advance 11.5.

### XET, MXE and CXE assays of FvXTH9 and FvXTH6

Endotransglucosylase assays were based on the method described by Fry *et al*. (1992). In brief, radiochemical XET activity assays were performed in a reaction volume of 64 μl that contained 100 mm succinate (Na^+^) buffer, pH 5.5, 0.5% (w/v) tamarind xyloglucan, 1 kBq [^3^H]XXXGol (giving a final concentration of 22 nm) and 4 μl of FvXTH enzyme extract. After incubation for various time periods at 25°C, the reaction was stopped by the addition of 20 μl 90% (v/v) formic acid. The reaction mixtures were then spotted on a 4 × 4‐cm square Whatman 3MM filter paper, air dried, washed under running tap water overnight and dried again in an oven at 60°C. For ^3^H scintillation counting, the dry paper was rolled into a cylinder with the loaded side facing outwards and placed into a 20‐ml scintillation vial, which was subsequently wetted with approximately 2 ml of scintillation cocktail (Wallac OptiPhase; Perkin Elmer, https://www.perkinelmer.com). The measurement of blanks was performed in an identical fashion.

Various acceptor substrates, including [^3^H]XXXGol, [^3^H]XXLGol, [^3^H]XLLGol, [^3^H]XXFGol and [^3^H]XXGol (each at 1 kBq per assay; thus final concentrations 22–820 nm), and also various donor substrates, such as tamarind xyloglucan, barley mixed‐linkage β‐glucan (MLG) and hydroxyethylcellulose (each at a final concentration of 0.5% (w/v) in the assay), were used to determine the substrate specificity of the enzymes. The impact of the pH level was investigated with reactions containing 1 kBq [^3^H]XXXGol as the acceptor substrate and 0.5% (w/v) of tamarind xyloglucan as the donor substrate. Different buffers were used as follows: 100 mm acetate buffer (Na^+^, pH 3.6, pH 4.0, pH 4.6 and pH 5.2), 100 mm succinate buffer (Na^+^, pH 5.0, pH 5.5, pH 6.0 and pH 6.5) and 100 mm phosphate buffer (Na^+^, pH 6.2, pH 7.0, pH 7.4 and pH 8.0). For MXE assays, the above method was used, but the donor substrate was 0.5% (w/v) medium‐viscosity barley MLG. CXE assays were performed in a final reaction volume of 30 μl that contained 100 mm succinate (Na^+^) buffer, pH 5.5, 0.33% (w/v) BSA, 0.17% (w/v) chlorobutanol, 2 kBq [^3^H]XXXGol (thus 93 nm XXXGol), 24 mg NaOH‐treated Whatman No. 1 cellulose (1.3 × 1.3 cm; 1.7 cm^2^) and 10 μl of FvXTH enzyme extract. The paper was thoroughly washed in water for 72 h and re‐washed in 6 m NaOH at 100°C for 30 min, then again under running tap water overnight and assayed for ^3^H, as in the XET assays.

### Kinetic assay of FvXTH6 and FvXTH9

TheXET kinetic parameters of the purified recombinant FvXTH9 and FvXTH6 were determined using radioactive assays, according to Fry *et al*. (1992). The *K*_m_ and *V*_max_ values for XXXGol were determined using 1% (w/v) tamarind xyloglucan as the donor substrate. As the acceptor substrate for XET activity assays, 1 kBq [^3^H]XXXGol of high specific activity (720 MBq μmol^−1^) diluted with various concentrations (0–357 μm) of unlabelled XXXGol was used. The *K*_m_ and *V*_max_ values for xyloglucan were determined using [^3^H]XXXGol at a concentration of 43 μm for FvXTH9 and 89 μm for FvXTH6, with various concentrations (0–4.55 mg ml^−1^) of the polysaccharide.

### Transient expression in *N. tabacum* leaves

To study the subcellular localization of FvXTH9 and FvXTH6 in *N. tabacum* leaves, full‐length genes of *FvXTH6* and *FvXTH9* were cloned into pGWR8 (Rozhon *et al*., 2010) plasmid and then fused with the YFP gene. Primers of FP_FvXTH9_pGWR8 and RP_FvXTH9_pGWR8 were used to amplify *FvXTH9*, whereas FP_FvXTH6_pGWR8 and RP_FvXTH6_pGWR8 were used to amplify *FvXTH6* (Table S2). The constructs were transformed into *A. tumefaciens* GV3101/pSoup cells. A single colony of recombinant *A. tumefaciens GV3101* was inoculated in 5 ml of Luria‐Bertani (LB) medium with kanamycin and rifampicin, and incubated at 28°C, 2000 ***g*** for overnight. Overnight culture (100 μL) was transferred into 20 ml of AB medium [1 g L^−1^ NH_4_Cl, 0.3 g L^−1^ MgSO_4_ × 7H_2_O, 0.15 g L^−1^ KCl, 0.01 g L^−1^ CaCl_2_ × 2H_2_O, 0.0025 g L^−1^ FeSO_4_ × 7H_2_O, 0.27 g L^−1^ KH_2_PO_4_, 10 g L^−1^ glucose, 3.90 g L^−1^ 2‐(*N*‐morpholino) ethanesulfonic acid (MES), adjusted with KOH to pH 5.6, sterilized by autoclaving and supplemented with acetosyringone to a final concentration of 100 μm) and incubated at 28°C, 2000 ***g*** overnight. The cell pellet was resuspended in infiltration buffer (10 mm MgSO_4_, 10 mm MES, 100 μm acetosyringone, pH 5.5) to an OD_600_ of 0.8 ± 0.1. The suspension was used for infiltration of the bottom side of tobacco leaves using a blunt‐ended 1‐ml syringe. After 3 days, the infiltration area was investigated using an Olympus FV1000/IX81 laser scanning confocal microscope with a UPlanSApo ×60/1.20 objective (Olympus, https://www.olympus-global.com) and laser wavelength of 515 nm. Images were obtained and processed using fluoview.

### Transient expression in *F. *×* ananassa* fruit

The PCR fragments cut by *Bam*HI and *Sac*I were cloned into the binary vector pBI121 digested with the same enzymes. This placed the insert under control of the CaMV *35S* promoter. Specific primers were used as follows: FP_FvXTH9_pBI121 and RP_FvXTH9_pBI121 for *FvXTH9* amplification; FP_FvXTH6_pBI121 and RP_FvXTH6_pBI121 for *FvXTH6* amplification (Table S2). The constructs were transformed into *E. coli* NEβ10 cells and the absence of errors confirmed by sequencing. Correct clones were transformed into *A. tumefaciens* AGL0. The *A. tumefaciens* strain AGL0 (Lazo *et al*., 1991) containing the recombinant pBI121 was grown at 28°C in LB medium with kanamycin and rifampicin antibiotics. When the culture reached an OD_600_ of about 0.8, *Agrobacterium* cells were harvested and washed twice with 30 ml MMA medium (Murashige and Skoog salts, 10 mm MES, pH 5.6, 20 g L^−1^ sucrose, according to Spolaore *et al*., 2001). Finally, the cell pellet was resuspended in 10 ml of MMA medium. The agrobacterium suspension was evenly injected into entire white fruits (14 days after pollination) that were still attached to the plant by using sterile 1‐ml hypodermic syringes. Infiltrated fruits were harvested on 8, 10, 12 and 14 dpi.

### Fruit firmness and LC_MS analysis

The firmness was measured using a texture analyser (TA.XT2i; Stable Micro Systems, https://www.stablemicrosystems.com) fitted with a 5‐mm flat probe. Each fruit was penetrated to 5 mm in depth at a speed rate of 0.5 mm sec^−1^ and the maximum force developed during the test was recorded in Newton (N). Each fruit was measured twice at opposite sides of its equatorial zone. LC‐MS was performed using an Agilent 1100 HPLC/UV system (Agilent Technologies, https://www.agilent.com) with a reverse‐phase column (Luna 3u C18(2) 100A, 150 × 2 mm; Phenomenex, https://www.phenomenex.com) and connected to a Bruker esquire3000plus ion‐trap mass spectrometer (Bruker, https://www.bruker.com). LC‐MS analysis was performed according to the protocol described by Ring *et al*. (2013). The values were expressed as per mil (‰) equivalent internal standard per dry weight using biochanin as internal standard.

## Accession numbers

FvXTH9, XP_004293486; FvXTH6, XP_004288290.

## Author contributions

This work was conceived and designed by LDW, WS, SCF and WR Experimental work was carried out and interpreted by LDW, SCF, TH, FCH and WR LDW, WS, SCF and WR contributed to data analysis and manuscript preparation.

## Conflict of interest

The authors declare no conflicts of interest.

## Supporting information


**Figure S1.** Expression levels of putative XTHs in *Fragaria vesca* varieties.
**Figure S2.** Phylogenetic tree of XTH candidate genes from *Fragaria vesca*.
**Figure S3.** qPCR analysis of FvXTH9 and FvXTH6.
**Figure S4.** Amino acid sequence alignment of group‐I/II XTHs.
**Figure S5.** Purification of FvXTH9‐His and FvXTH6‐His.
**Figure S6.** pH optima of FvXTH9 and FvXTH6.
**Figure S7**. Metabolite analysis of *Fragaria* × *ananassa* fruit after agroinfiltration.
**Figure S8**. Amino acid sequence alignment.

**Table S1.** Prediction of the subcellular localization of FvXTH6 and FvXTH9.
**Table S2.** List of primers.
