# Supplementary material for: Higher expression of the strawberry xyloglucan endotransglucosylase/hydrolase genes FvXTH9 and FvXTH6 accelerates fruit ripening
Source: Plant J. 2019 Oct 8;100(6):1237–53. doi: 10.1111/tpj.14512 (PMC8653885; doi:10.1111/tpj.14512)
Supplement: Supplementary file 1 — Figure S1. Expression levels of putative XTHs in Fragaria vesca varieties. Figure S2. Phylogenetic tree of XTH candidate genes from Fragaria vesca. Figure S3. qPCR analysis of FvXTH9 and FvXTH6. Figure S4. Amino acid sequence alignment of group‐I/II XTHs. Figure S5. Purification of FvXTH9‐His and FvXTH6‐His. Figure S6. pH optima of FvXTH9 and FvXTH6. Figure S7. Metabolite analysis of Fragaria × ananassa fruit after agroinfiltration. Figure S8. Amino acid sequence alignment. [file TPJ-100-1237-s002.pdf]

SUPPLEMENTAL MATERIAL
Figures

Higher expression of the strawberry xyloglucan endotransglucosylase/hydrolase genes *FvXTH9* and *FvXTH6* accelerates fruit ripening

Lucia D. Witasari[1,2], Fong-Chin Huang[1], Thomas Hoffmann[1], Wilfried Rozhon[3], Stephen C. Fry[4], Wilfried Schwab[1]

[1] Biotechnology of Natural Products, Technische Universität München, Liesel-Beckmann-Str. 1, 85354 Freising, Germany

[2] Department of Food and Agricultural Product Technology, Faculty of Agricultural Technology, Universitas Gadjah Mada, Jl. Flora No. 1 – Bulaksumur Yogyakarta, Indonesia

[3] Biotechnology of Horticultural Crops, TUM School of Life Sciences Weihenstephan, Technische Universität München, Liesel-Beckmann-Str. 1, 85354 Freising, Germany

[4] Edinburgh Cell Wall Group, Institute of Molecular Plant Sciences, The University of Edinburgh, Daniel Rutherford Building, The King's Buildings, Edinburgh EH9 3BF, UK

Figure S1. Expression levels of putative XTHs in *Fragaria vesca* varieties.

Figure S2. Phylogenetic tree of XTH candidate genes from *F. vesca.*

Figure S3. qPCR analysis of FvXTH9 and FvXTH6.

Figure S4. Amino acid sequence alignment of group I/II XTHs.

Figure S5. Purification of FvXTH9-His and FvXTH6-His.

Figure S6. pH optimum of FvXTH9 and FvXTH6.

Figure S7. Metabolite analysis of *F.* × *ananassa* fruit after agroinfiltration.

Figure S8. Amino acid sequence alignment.

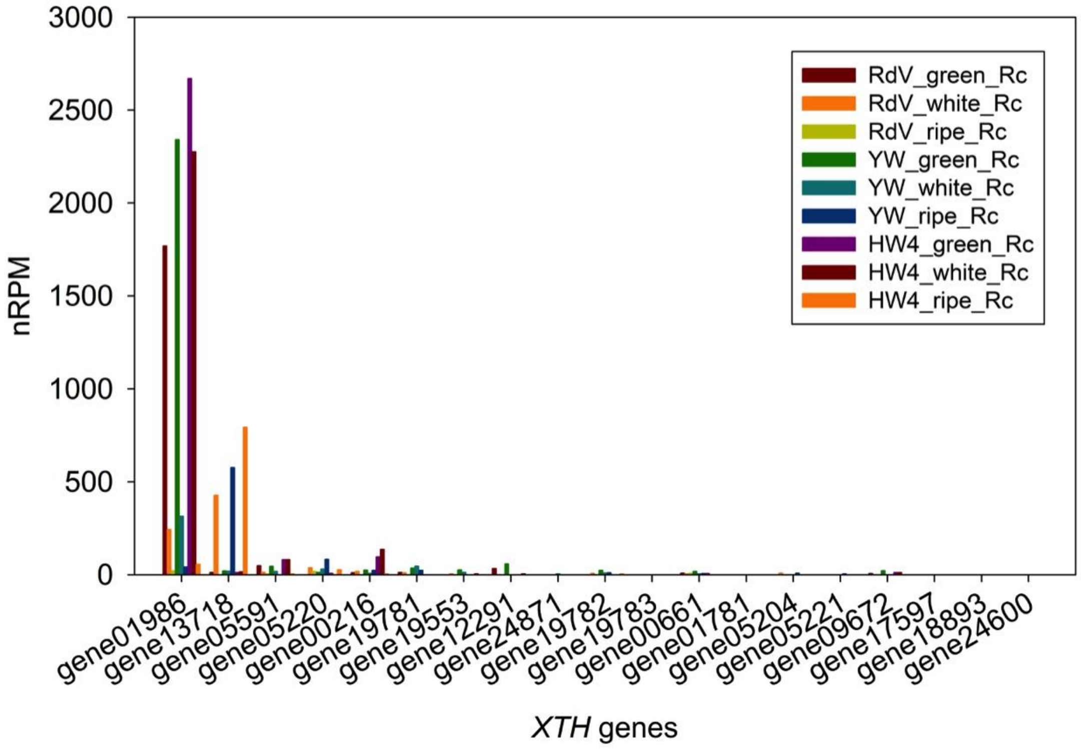

Figure S1. Expression levels of putative XTHs in *Fragaria vesca* varieties Reine de Vallées, Hawaii 4 and Yellow Wonder.

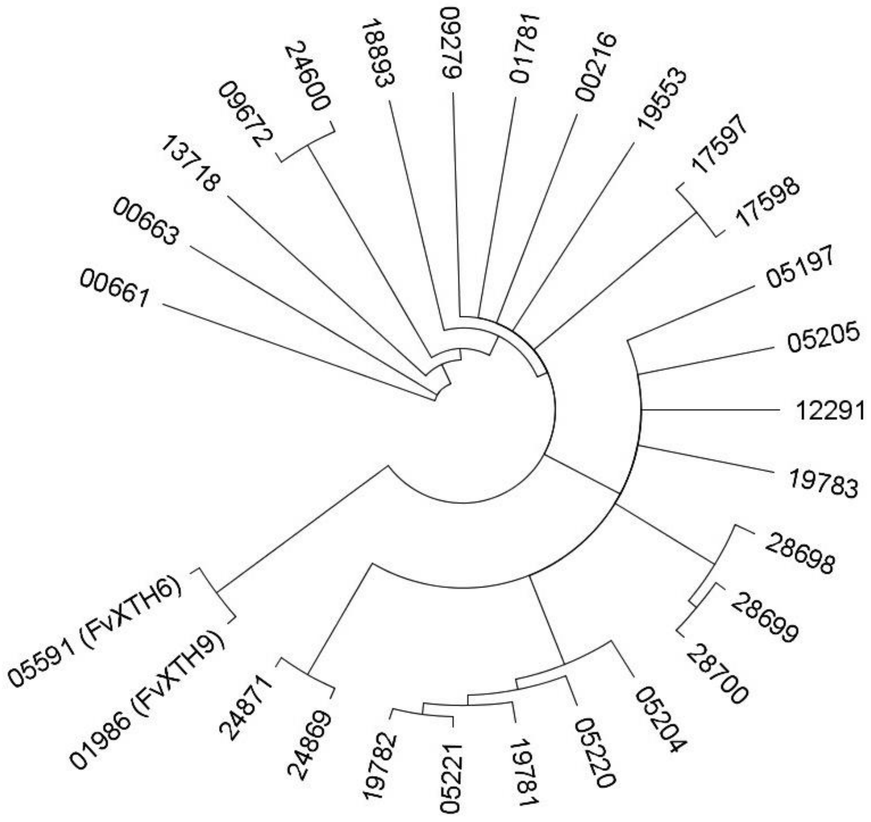

Figure S2. Phylogenetic tree of *XTH* candidate genes from *F. vesca*. The tree was constructed using Genious v5.6 software with the Neighbor-Joining method with 5000 bootstrap replications. Amino acid sequences were retrieved from the *F. vesca* Genome Browser database and their accession numbers are as follows: gene00216, gene00661, gene00663, gene01781, gene01986, gene05197, gene05204, gene05205, gene05220, gene05221, gene05591, gene09279, gene09672, gene12291, gene13718, gene17597, gene17598, gene18893, gene19553, gene19781, gene19782, gene19783, gene24600, gene24869, gene24871, gene28698, gene28699, and gene28700.

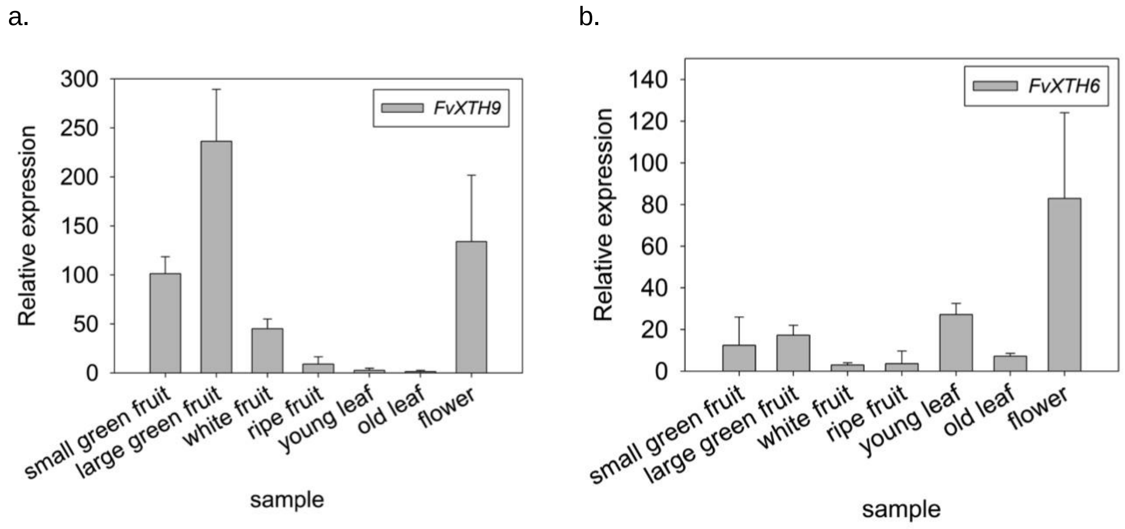

Figure S3. qPCR analysis of *FvXTH9* (a) and *FvXTH6* (b) at different fruit maturation stages (small green, large green, white and ripe fruit) as well as other tissues (young leaf, old leaf and flower) of *F. vesca.* Hawaii 4. Relative expression levels are normalized to the reference gene (interspacer gene).

```
                                                                     Section 1
                (1) 1        10        20        30        40             55
 At-XTH10       (1) ----MTLINRSKPFVLLVGFSIISSLLLWVSQASVVSSGDFNKDFFVTWSPTHVN
 At-XTH6        (1) -----MAKIYSP-SFPGTLCLCIFTLLTLMFIRVSARPATFVEDFKAAWSESHIR
 At-XTH7        (1) ----MVVSLFSSRNVFYTLSLCLFAAL---YQPVMSRPAKFEDDFRIAWSDTHIT
 FvXTH6         (1) ----MYPSLRSG-SVIASISLCFLSLFS---LSAFARPATFLQDFQVTWSDSHIR
 Sl-XTH7        (1) MATLTCCSLKNS-AFVLILVYALTFSFS---LVS-ARPATFLQDFKIAWSDSHIK
 At-XTH9        (1) ---------MVGMDLFKCVMMIMV---LVVSCGEAVSGAKFDELYRSSWAMDHCV
 FvXTH9         (1) ---------MASASLFLSVILGLS---LFLG---PVSSAKFDELFQPYWASDHFT
 At-XTH8        (1) --METERRIITSCSAMTALFLFMTALMASSSIAATPTQS-FEDNFNIMWSENHFT
 Sl-XTH1        (1) --------MGIIKGVLFSIVLINLSLVVFCGYPRRPVDVPFWKNYEPSWASHHIK
 Sl-XTH4        (1) -----------MKGVLVAFVLINLSILASCGAPRKVIDVPFWNNYEPSWSSHHIK
 At-XTH4        (1) ------MTVSSSPWALMALFLMVSSTMVMAIPPRKAIDVPFGRNYVPTWAFDHQK
 At-XTH5        (1) ------MG-RLSSTLCLTFLILAT--VAFGVPPKKSINVPFGRNYFPTWAFDHIK
 Fc-XTH1        (1) ------MASSQQCTLLLSIMLMGS--VTMAAPPKRPVSVPFGRNYMPTWAFDHIK
 Md-XTH1        (1) ------MASCKQWTVFLSLLCLVS--ATVAAPPKKPVAVPFGRNYMPTWAFDHIK
 PttXET16A      (1) ------MAAAYPWTLFLGMLVMVS--GTMGAALRKPVDVAFGRNYVPTWAFDHIK
 Consensus      (1)              SVLLSLLLILS   L                F   NY PTWA   HIK
                                                                     Section 2
               (56) 56            70        80        90        100     110
 At-XTH10      (52) TSNDGRSRTLKLDQESGASFSSIQTFLFGQIDMKIKLIRGSSQGTVVAYYMSSDQ
 At-XTH6       (50) QMEDGKAIQLVLDQSTGCGFASKRKYLFGRVSMKIKLIPGDSAGTVTAFYMNSDT
 At-XTH7       (49) QIDGGRAIQLKLDPSSGCGFASKKQYLFGRVSMKIKLIPGDSAGTVTAFYMNSDT
 FvXTH6        (48) QIDGGRAIQLVLDQNSGCGFSSKHKYLFGRVSMKIKLIPGDSAGTVTAFYMNSDT
 Sl-XTH7       (51) QLDGGRGIQLILDQNSGCGFASRSKYLFGRVSMKIKLVPGDSAGTVTAFYMNSDT
 At-XTH9       (44) N--EGEVTKLKLDNYSGAGFESRSKYLFGKVSIQIKLVEGDSAGTVTAFYMSSDG
 FvXTH9        (41) Y--EGELLHMKLDNYSGAGFSSKNKYMFGKVTVQIKLVEGDSAGTVTAFYMSSDG
 At-XTH8       (53) TSDDGEIWNLSLDNDTGCGFQTKHMYRFGWFSMKLKLVGGDSAGVVTAYYMCSEN
 Sl-XTH1       (48) FLNGGTTTDLILDRSSGAGFQSKKSYLFGHFSMKMRLVGGDSAGVVTAFYLSSNN
 Sl-XTH4       (45) YLNGGTTAELLLDKSSGTGFQSKRSYLFGHFSMKMKLVGGDSAGVVTAFYLSSTN
 At-XTH4       (50) QFNGGSELQLILDKYTGTGFQSKGSYLFGHFSMHIKLPAGDTAGVVTAFYLSSTN
 At-XTH5       (47) YLNGGSEVHLVLDKYTGTGFQSKGSYLFGHFSMHIKMVAGDSAGTVTAFYLSSQN
 Fc-XTH1       (48) YFNGGNEIQLHLDKYTGTGFQSKGSYLFGHFHMQIKLPPGDSAGTVTAFYLSSTN
 Md-XTH1       (48) YFNGGKEIQLHLDKYTGTGFQSKGNYLFGHFHMQIKMVPGDSAGTVTAYYLSSQN
 PttXET16A     (48) YFNGGNEIQLHLDKYTGTGFQSKGSYLFGHFSMQMKLVPGDSAGTVTAFYLSSQN
 Consensus     (56)     NGG   IQL LD   SG GFQSK  YLFG FSMKIKLV GDSAGTVTAFYMSSDN
                                                                     Section 3
              (111) 111        120  ★★★130        140       150           165
 At-XTH10     (107) ---PNRDEIDFEFLGNVNGQPYILQTNVYAEGLDNREERIHLWFDPAKDFHTYSI
 At-XTH6      (105) --ATVRDELDFEFLGNRSGQPYSVQTNIFAHGKGDREQRVNLWFDPSMDYHTYTI
 At-XTH7      (104) --DSVRDELDFEFLGNRSGQPYTVQTNVFAHGKGDREQRVNLWFDPSRDFHEYAI
 FvXTH6       (103) --DAVRDELDFEFLGNRTGQPYTVQTNIYAHGQGNREERVNLWFDPAADFHTYTI
 Sl-XTH7      (106) --DNVRDELDFEFLGNRTGQPYTVQTNVYVHGKGDKEQRVNLWFDPSADFHTYTI
 At-XTH9       (97) ---PNHNEFDFEFLGNTTGEPYIVQTNIYVNGVGNREQRLNLWFDPTTEFHTYSI
 FvXTH9        (94) ---PLHNEFDFEFLGNTTGEPYSVQTNLYINGVGNREQRLDLWFDPTTDFHSYSI
 At-XTH8      (108) GAGPERDEIDFEFLGNRTGQPYIIQTNVYKNGTGNREMRHSLWFDPTKDYHTYSI
 Sl-XTH1      (103) ---AEHDEIDFEFLGNRTGQPYILQTNVFTGGKGNREQRIYLWFDPTKGYHSYSV
 Sl-XTH4      (100) ---AEHDEIDFEFLGNRTGQPYILQTNVFTGGKGDREQRIYLWFDPTKDFHSYSV
 At-XTH4      (105) ---NEHDEIDFEFLGNRTGQPAILQTNVFTGGKGNREQRIYLWFDPSKAYHTYSI
 At-XTH5      (102) ---SEHDEIDFEFLGNRTGQPYILQTNVFTGGAGNREQRINLWFDPSKDYHSYSV
 Fc-XTH1      (103) ---AEHDEIDFEFLGNRTGQPFILQTNVFTGGKGDREQRIFLWFDPTKEYHSYSV
 Md-XTH1      (103) ---NEHDEIDFEFLGNRTGQPYILQTNVFTGGKGDREQRIFLWFDPTAAYHSYAV
 PttXET16A    (103) ---SEHDEIDFEFLGNRTGQPYILQTNVFTGGKGDREQRIYLWFDPTKEFHYYSV
 Consensus    (111)       EHDEIDFEFLGNRTGQPYILQTNVF   GKGNREQRI LWFDPTKDFHTYSI
```

```
                                                                          Section 4
          (166) 166           180       190       200       210       220
 At-XTH10 (159) LWNIHQIVFMVDQIPIRLYRNHGEKGVA--YPRLQPMSVQASLWNGESWATRGGH
  At-XTH6 (158) LWSHKHIVFYVDDVPIREYKNNEAKNIA--YPTSQPMGVYSTLWEADDWATRGGL
  At-XTH7 (157) SWNHLRIVFYVDNVPIRVYKNNEARKVP--YPRFQPMGVYSTLWEADDWATRGGI
   FvXTH6 (156) LWNHHHIVFYVDDVPIRLYKNNEAKGIP--YPKLQPMGVFSTLWEADDWATRGGL
  Sl-XTH7 (159) FWNHHQAVFSVDGIPIRVYKNNEAKGIP--FPKFQPMGVYSTLWEADDWATRGGL
  At-XTH9 (149) LWSKRSVVFMVDETPIRVQKNLEEKGIP--FAKDQAMGVYSSIWNADDWATQGGL
   FvXTH9 (146) FWNQRQVVFLVDETPIRVHTNMESKGLP--FPKDQAMGVYSSIWNADDWATQGGR
  At-XTH8 (163) LWNNHQLVFFVDRVPIRVYKNSDKVPNNDFFPNQKPMYLFSSIWNADDWATRGGL
  Sl-XTH1 (155) LWNTYLIVIFVDDVPIRAFKNSKDLGVK--FPFNQPMKIYSSLWDADDWATRGGL
  Sl-XTH4 (152) LWNTYQIAIFVDDVPIRVFKNSKDIGVK--FPFNQPMKIYSSLWNADDWATRGGL
  At-XTH4 (157) LWNMYQIVFFVDNIPIRTFKNAKDLGVR--FPFNQPMKLYSSLWNADDWATRGGL
  At-XTH5 (154) LWNMYQIVFFVDDVPIRVFKNSKDVGVK--FPFNQPMKIYSSLWNADDWATRGGL
  Fc-XTH1 (155) LWNLYQIVFFVDDIPIRVFKNSKDLGVK--FPFNQPMKLYSSLWNADDWATRGGL
  Md-XTH1 (155) LWNLYQIVFLVDDIPIRVFKNSKDLGVK--FPFNQPMKLYSSLWNADDWATRGGL
PttXET16A (155) LWNMYMIVFLVDDVPIRVFKNCKDLGVK--FPFNQPMKIYSSLWNADDWATRGGL
Consensus (166) LWN YQIVFFVDDVPIRVFKN  DLGV   FP NQPM VYSSLWNADDWATRGGL
                                                                          Section 5
          (221) 221           230       240       250       260           275
 At-XTH10 (212) DKIDWSKGPFVASFGDYKIDACIWIGNTSFCN--------GESTENWWNKNEFSS
  At-XTH6 (211) EKIDWSKAPFYAYYKDFDIEGCPVPGPTF----------CPSNPHNWWEGYAYQS
  At-XTH7 (210) EKINWSRAPFYAYYKDFDIEGCPVPGPAD----------CPANSKNWWEGSAYHQ
   FvXTH6 (209) EKINWSKAPFYAYYKDFDIEGCSVPGPAN----------CASSAQNWWEGTAYQA
  Sl-XTH7 (212) EKINWSKSPFFAYYKDFDIEGCAMPGPAN----------CASNPSNWWEGPAYQQ
  At-XTH9 (202) VKTDWSHAPFVASYKEFQIDACEIPTTTD-------LSKCNGDQKFWWDEPTVSE
   FvXTH9 (199) VKTDWSHGPFVASYKGFDINACECPASVAGAENAKKCSSSNGDKKYWWDEPVLSE
  At-XTH8 (218) EKTDWKKAPFVSSYKDFAVEGCRWKDPFPACV-------STTTEN-WWDQYDAWH
  Sl-XTH1 (208) EKTNWANAPFTASYTSFHVDGCEAATPQEVQV-------CNTKGMKWWDQKAFQD
  Sl-XTH4 (205) EKTNWSGAPFIASYTSFHIDGCEAVTPQEVQV-------CNTNGMKWWDQKAFQD
  At-XTH4 (210) EKTNWANAPFVASYKGFHIDGCQAS--VEAKY-------CATQGRMWWDQKEFRD
  At-XTH5 (207) EKTNWEKAPFVASYRGFHVDGCEAS--VNAKF-------CETQGKRWWDQKEFQD
  Fc-XTH1 (208) EKTDWSKAPFVATYRGFHIDGCEAS--VQARF-------CATQGKRWWDQKEFQD
  Md-XTH1 (208) EKTDWSKAPFIASYRGFHIDGCEAS--VEAKY-------CATQGKRWWDQKEFQD
PttXET16A (208) EKTDWSKAPFIASYRSFHIDGCEAS--VEAKF-------CATQGARWWDQKEFQD
Consensus (221) EKTDWSKAPFVASYK F IDGCE                C TQG  WWDQ  FQD
                                                                          Section 6
          (276) 276           290       300                317
 At-XTH10 (259) LTRVQKRWFKWVRKYHLIYDYCQDYGRFNNKLPKECSLPKY-
  At-XTH6 (256) LNAVEARRYRWVRVNHMVYDYCTDRSRFP-VPPPECRA----
  At-XTH7 (255) LSPVEARSYRWVRVNHMVYDYCTDKSRFP-VPPPECSAGI--
   FvXTH6 (254) LNALEYRRYKWVRMNHMIYDYCSDRSRYP-KPPPECVAGL--
  Sl-XTH7 (257) LSPVQARQYRWVRMNHMIYDYCTDKSRNP-VPPPECRAGI--
  At-XTH9 (250) LSLHQNHQLIWVRANHMIYDYCFDATRFP-VTPLECQHHRHL
   FvXTH9 (254) LNVHQNHQLVWVKNHHMVYDYCTDSARFP-VTPVECVHHRH-
  At-XTH8 (265) LSKTQKMDYAWVQRNLVVYDYCKDSERFP-TLPWECSISPWA
  Sl-XTH1 (256) LDALQYRRLRWVRQKYTVYNYCTDKARYP-VPPPECTKDRDI
  Sl-XTH4 (253) LDGPEYRKLHRVRQNFXIYNYCTDRKRYP-TLPLECTRDRDL
  At-XTH4 (256) LDAEQWRRLKWVRMKWTIYNYCTDRTRFP-VMPAECKRDRDA
  At-XTH5 (253) LDANQYKRLKWVRKRYTIYNYCTDRVRFP-VPPPECRRDRDI
  Fc-XTH1 (254) LDAYQWRRLRWVRQRFTIYNYCTDRTRYP-TLPAEVQRDRDI
  Md-XTH1 (254) LDAQQWRRLRWVRKKFTIYNYCTDRVRYP-SMPPECKRDRDI
PttXET16A (254) LDAFQYRRLSWVRQKYTIYNYCTDRSRYP-SMPPECKRDRDI
Consensus (276) L A QYRRLRWVR  HMIYDYCTDRSRFP V PPEC   R
```

Figure S4. Amino acid sequence alignment of group I/II XTHs using AlignX Vector NTI Advance V.11.5. software. Sequences correspond to the following GenBank accession numbers: *A. thaliana* (At-XTH4 (NP 178708), At-XTH5 (NP 196891), At-XTH6 (NP 569019), At-XTH7 (NP 195494), At-XTH8 (NP 563892), At-XTH9 (NP 192230), At-XTH10 (NP 179069), *S. lycopersicum* (Sl-XTH1, BAA03923), Sl-XTH4 (AAG43444), Sl-XTH7 (AAS46243), *M. × domestica* (Md-XTH1, AAN07897), *F. chiloensis* (Fc-XTH1, ADE42488) and *Populus tremula* × *Populus tremuloides* (PttXET16A, AAN87142). Identical amino acids in the sequences are shaded in black. The red box indicates the conserved motif and asteriks (***) mark the N-linked glycosylation motif.

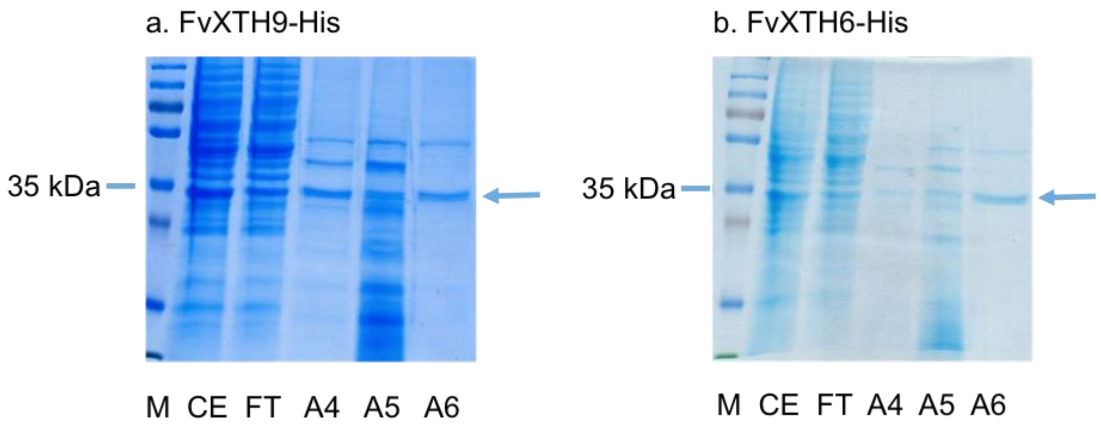

Figure S5. Purification of FvXTH9-His and FvXTH6-His. M, protein marker; CE, crude extract; FT, flow through; A4-, A5-, and A6-fractions were eluted with 250 mM imidazole. Target protein (33 kDa) is shown by arrow.

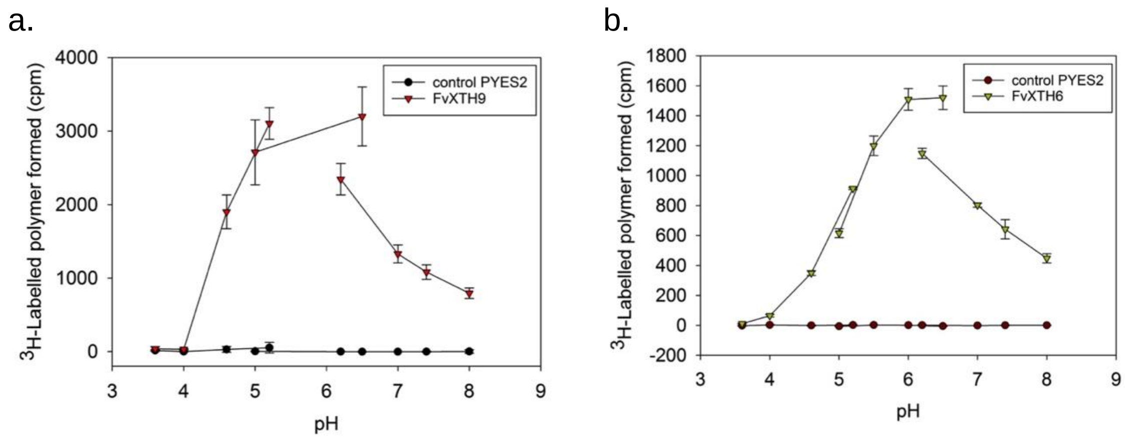

Figure S6. pH optimum of FvXTH9 (a) and FvXTH6 (b). No XET activity was present in control PYES2. Different buffers were used as follows: sodium acetate buffer (pH 3.6, pH 4.0, pH 4.6, and pH 5.2), sodium succinate buffer (pH 5.0, pH 5.5, pH 6.0, and pH 6.5) and sodium phosphate buffer (pH 6.2, pH 7.0, pH 7.4, and pH 8.0). Each assay was performed as 4 replicates; error bars are standard deviation

a.

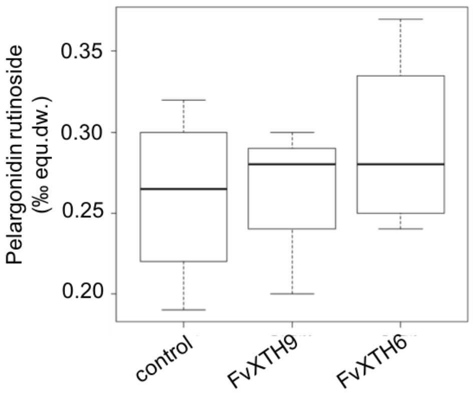
Pelargonidin rutinoside
(‰ equ.dw.)
0.35
0.30
0.25
0.20
control
FvXTH9
FvXTH6

b.

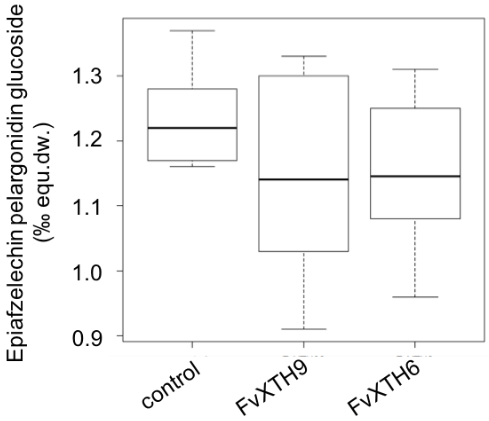
Epiafzelechin pelargonidin glucoside
(‰ equ.dw.)
1.3
1.2
1.1
1.0
0.9
control
FvXTH9
FvXTH6

c.

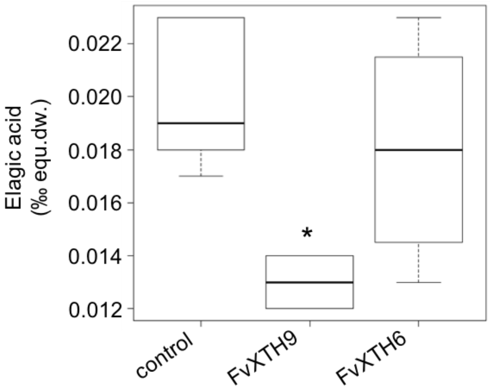
Elagic acid
(‰ equ.dw.)
0.022
0.020
0.018
0.016
0.014
0.012
*
control
FvXTH9
FvXTH6

d.

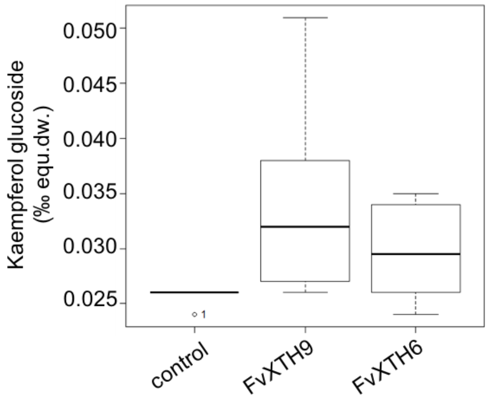
Kaempferol glucoside
(‰ equ.dw.)
0.050
0.045
0.040
0.035
0.030
0.025
1
control
FvXTH9
FvXTH6

e.

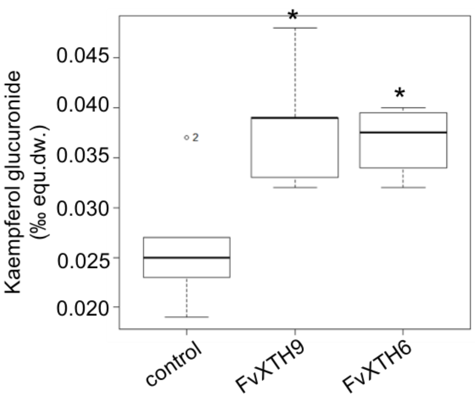
Kaempferol glucuronide
(‰ equ.dw.)
0.045
0.040
0.035
0.030
0.025
0.020
2
*
*
control
FvXTH9
FvXTH6

f.

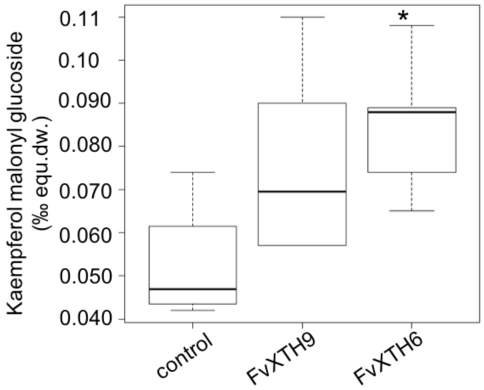
Kaempferol malonyl glucoside
(‰ equ.dw.)
0.11
0.10
0.090
0.080
0.070
0.060
0.050
0.040
*
control
FvXTH9
FvXTH6

g.

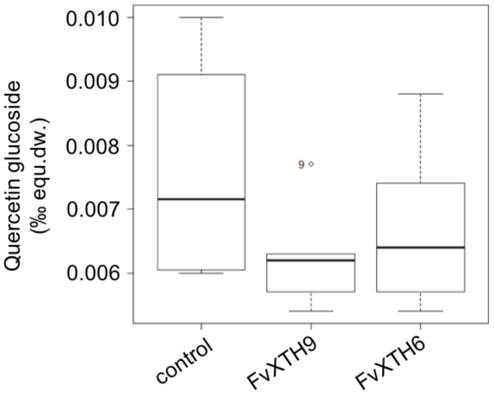
Quercetin glucoside
(‰ equ.dw.)
0.010
0.009
0.008
0.007
0.006
9
control
FvXTH9
FvXTH6

h.

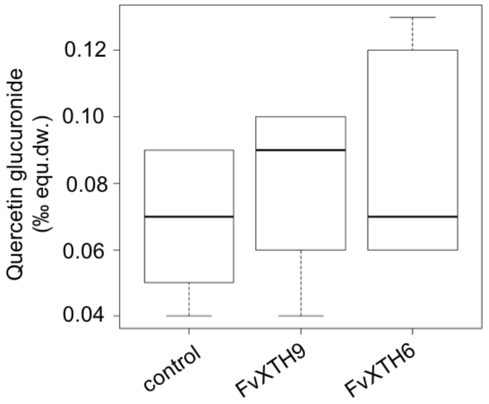
Quercetin glucuronide
(‰ equ.dw.)
0.12
0.10
0.08
0.06
0.04
control
FvXTH9
FvXTH6

i.

j.

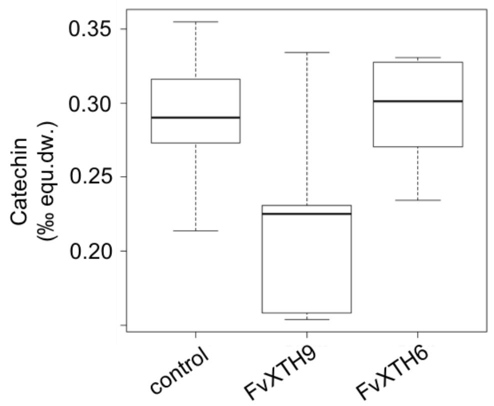

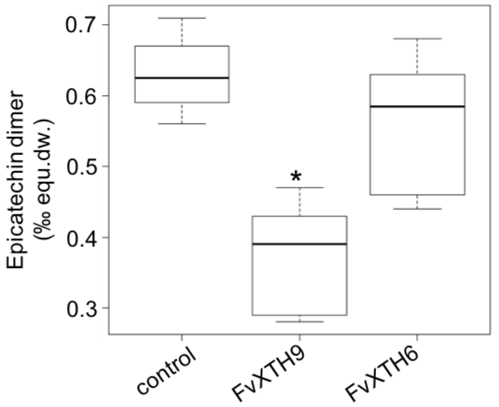

k.

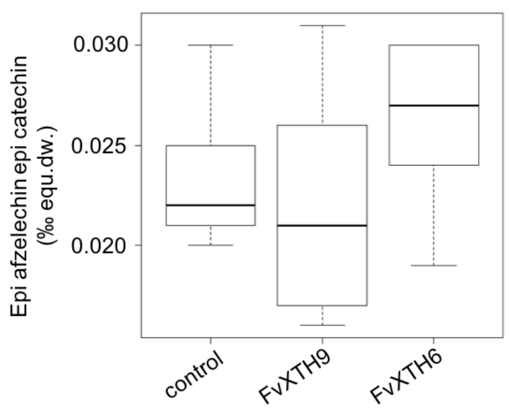

l.

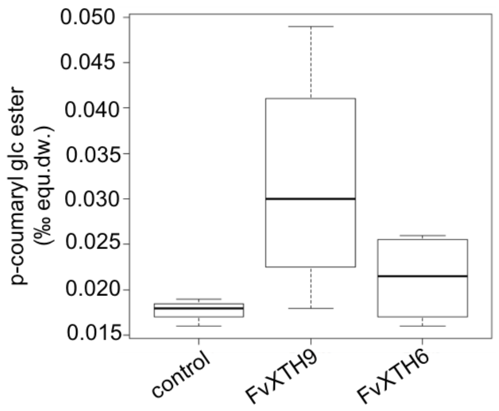

m.

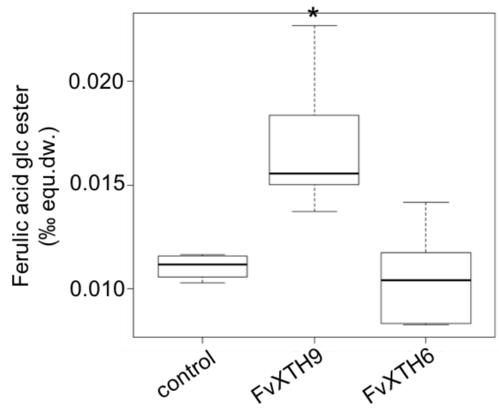

n.

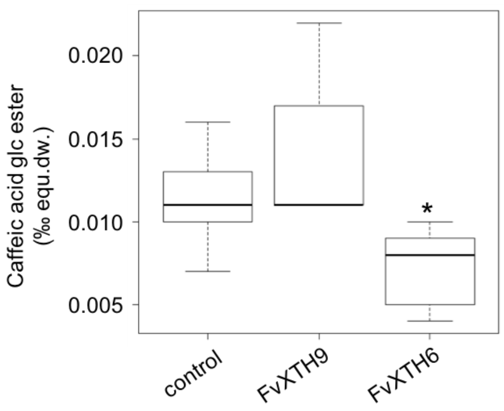

Figure S7. Metabolite analysis of *F.* × *ananassa* fruit after agroinfiltration. Relative concentration (‰ equivalent of internal standard of the dry weight) of (a) pelargonidin rutinoside, (b) epiafzelechin pelargonidin glucoside, (c) ellagic acid, (d) kaempferol glucoside, (e) kaempferol glucuronide, (f) kaempferol malonyl glucoside, (g) quercetin glucoside, (h) quercetin glucuronide, (i) catechin, (j) epicatechin dimer, (k) epiafzelechin-epicatechin, (l) p-coumaryl glc ester, (m) ferulic acid glc ester, and (n) caffeic acid glc ester. The data were obtained by analysing 4-6 fruits (10 DPI) for each sample. Control fruit was infiltrated with *A. tumefaciens* AgL0 contained pBI121 empty plasmid. The asterisk indicates statistically significant differences ($p < 0.05$) between agroinfiltrated fruits with *XTHs* and the empty plasmid.

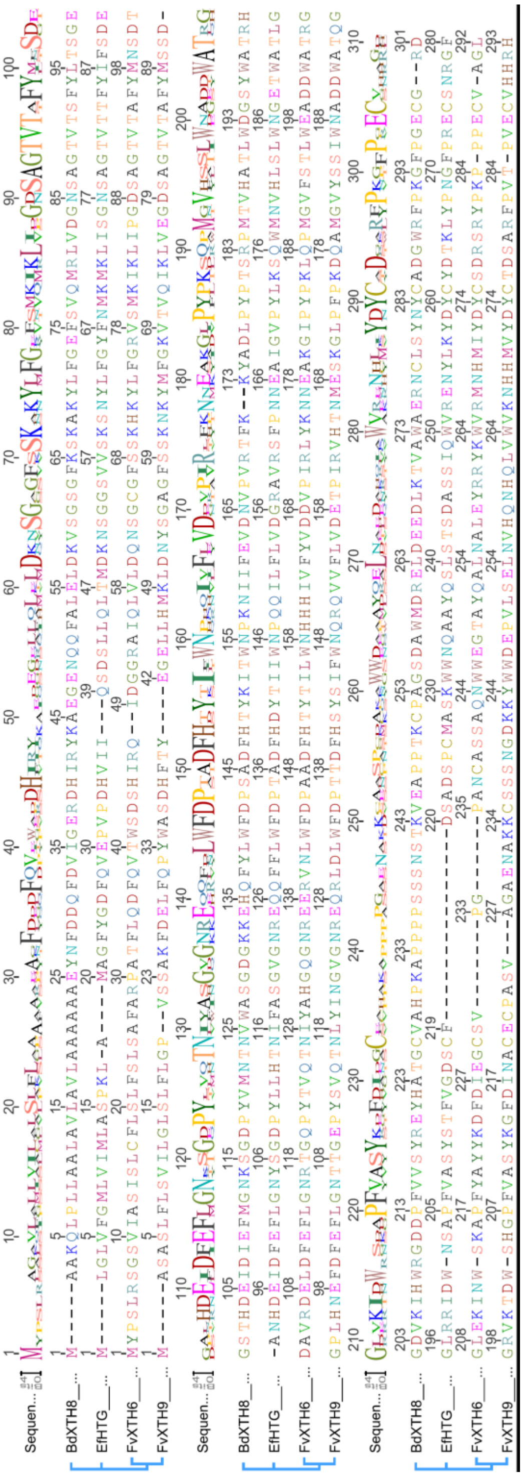

	BdXTH8	EfHTG	FvXTH6	FvXTH9
BdXTH8	100	38.2	35.5	35.6
EfHTG	38.2	100	44.6	39.1
FvXTH6	35.5	44.6	100	45.7
FvXTH9	35.6	39.1	45.7	100

Figure S8. Amino acid sequence alignment of BdXTH8 (XP_003573584.1), EfHTG (CEH24720.1), FvXTH6 (gene05591), and FvXTH9 (gene01986) as well as pairwise sequence identities using Geneious software with default values.
