# Supplementary material for: Higher expression of the strawberry xyloglucan endotransglucosylase/hydrolase genes FvXTH9 and FvXTH6 accelerates fruit ripening
Source: Plant J. 2019 Oct 8;100(6):1237–53. doi: 10.1111/tpj.14512 (PMC8653885; doi:10.1111/tpj.14512)
Supplement: Supplementary file 2 — Table S1. Prediction of the subcellular localization of FvXTH6 and FvXTH9. Table S2. List of primers. [file TPJ-100-1237-s001.pdf]

SUPPLEMENTAL MATERIAL TABLES

Higher expression of the strawberry xyloglucan endotransglucosylase/hydrolase genes *FvXTH9* and *FvXTH6* accelerates fruit ripening

Lucia D. Witasari[1,2], Fong-Chin Huang[1], Thomas Hoffmann[1], Wilfried Rozhon[3], Stephen C. Fry[4], Wilfried Schwab[1]

[1] Biotechnology of Natural Products, Technische Universität München, Liesel-Beckmann-Str. 1, 85354 Freising, Germany

[2] Department of Food and Agricultural Product Technology, Faculty of Agricultural Technology, Universitas Gadjah Mada, Jl. Flora No. 1 – Bulaksumur Yogyakarta, Indonesia

[3] Biotechnology of Horticultural Crops, TUM School of Life Sciences Weihenstephan, Technische Universität München, Liesel-Beckmann-Str. 1, 85354 Freising, Germany

[4] Edinburgh Cell Wall Group, Institute of Molecular Plant Sciences, The University of Edinburgh, Daniel Rutherford Building, The King's Buildings, Edinburgh EH9 3BF, UK

Table S1. Prediction of the sub-cellular localization of FvXTH6 and FvXTH9.

Table S2. List of primers.

Table S1. Prediction of the sub-cellular localization of FvXTH9 and FvXTH6.

Prediction tool	Reference	Results for FvXTH6	Results for FvXTH9
Predotar 1.04	(Small *et al.,* 2004)	ER (probability: 0.89)	ER (probability: 0.99)
TargetP 1.1	(Emanuelsson *et al.,* 2000)	Secretory pathway (probability: 0.958)	Secretory pathway (probability: 0.974)
Plant-mPLoc	(Chou and Shen, 2007, 2008, 2010)	Cell wall	Cell wall
MultiLoc2	(Blum *et al.,* 2009)	Secretory pathway (probability: 1.0)	Secretory pathway (probability: 0.99)

Table S2. List of primers.

Primer	Sequence
FP_FvXTH9_qPCR	5’-CGC TGA CGA CTG GGC CAC AC-3'
RP_FvXTH9_qPCR	5’-GCC GGG CAC TCA CAG GCA TT-3'
FP_FvXTH6_qPCR	5’-GAG GCA GGG CCA TCC AGC TC-3'
RP_FvXTH6_qPCR	5’-TCC GGC AGA GTC ACC GGG AA-3'
IS-RT_F	5’-ACC GTT GAT TCG CAC AAT TGG TCA TCG-3'
IS-RT_R	5’-TAC TGC GGG TCG GCA ATC GGA CG-3'
FP_FvXTH9_PYES2	5’-CGG GGT ACC AAC ACA ATG TCT TCT GCC TCT TTG-3’
RP_FvXTH9HIS_PYES2	5’-TAA AGC GGC CGC TTA ATG ATG ATG ATG ATG ATG GTG ACG GTG GTG C-3’.
FP_FvXTH6_PYES2	5'-CGG AAT TCA ACA CAA TGT CTC CCT CTT TG-3'
RP_FvXTH6HIS_PYES2	5'-TAA AGC GGC CGC TTA ATG ATG ATG ATG ATG ATG GAG GCC GGC GAC A-3'
FP_FvXTH9_pGWR8	5’-ACC GGA TAT CAT GGC TTC TGC CTC TTT G-3’
RP_FvXTH9_pGWR8	5’-TAT AGC GGC CGC TGT GAC GGT GGT G-3’
FP_FvXTH6_pGWR8	5’-CAT GAC ATG TAT CCC TCT TTG AGG AG-3’
RP_FvXTH6_pGWR8	5’-TAT AGC GGC CGC TGA GGC CGG CGA C-3’
FP_FvXTH9_pBI121	5’-TAG AGG ATC CAT GGC TTC TGC CTC-3’
RP_FvXTH9_pBI121	5’-ATT CGA GCT CCT AGT GAC GGT GGT GC-3’,
FP_FvXTH6_pBI121	5’-TAG AGG ATC CAT GTA TCC CTC TTT G-3’
RP_FvXTH6_pBI121	5’-ATT CGA GCT CTT AGA GGC CGG CGA C-3’
