# Supplementary material for: Higher expression of the strawberry xyloglucan endotransglucosylase/hydrolase genes FvXTH9 and FvXTH6 accelerates fruit ripening
Source: Plant J. 2019 Oct 8;100(6):1237–53. doi: 10.1111/tpj.14512 (PMC8653885; doi:10.1111/tpj.14512)
Supplement: Supplementary file 3 [file TPJ-100-1237-s003.docx]

TPJ-00865-2019.R1_Supporting_Information_Legends

Figure S1. Expression levels of putative XTHs in *Fragaria vesca* varieties Reine de Vallées, Hawaii 4 and Yellow Wonder.

Figure S2. Phylogenetic tree of *XTH* candidate genes from *F. vesca*. The tree was constructed using Genious v5.6 software with the Neighbor-Joining method with 5000 bootstrap replications. Amino acid sequences were retrieved from the *F. vesca* Genome Browser database and their accession numbers are as follows: gene00216, gene00661, gene00663, gene01781, gene01986, gene05197, gene05204, gene05205, gene05220, gene05221, gene05591, gene09279, gene09672, gene12291, gene13718, gene17597, gene17598, gene18893, gene19553, gene19781, gene19782, gene19783, gene24600, gene24869, gene24871, gene28698, gene28699, and gene28700.

Figure S3. qPCR analysis of *FvXTH9* (a) and *FvXTH6* (b) at different fruit maturation stages (small green, large green, white and ripe fruit) as well as other tissues (young leaf, old leaf and flower) of *F. vesca.* Hawaii 4. Relative expression levels are normalized to the reference gene (interspacer gene).

Figure S4. Amino acid sequence alignment of group I/II XTHs using AlignX Vector NTI Advance V.11.5. software. Sequences correspond to the following GenBank accession numbers: *A. thaliana* (At-XTH4 (NP 178708), At-XTH5 (NP 196891), At-XTH6 (NP 569019), At-XTH7 (NP 195494), At-XTH8 (NP 563892), At-XTH9 (NP 192230), At-XTH10 (NP 179069), *S. lycopersicum* (Sl-XTH1, BAA03923), Sl-XTH4 (AAG43444), Sl-XTH7 (AAS46243), *M. × domestica* (Md-XTH1, AAN07897), *F. chiloensis* (Fc-XTH1, ADE42488) and *Populus tremula × Populus tremuloides* (PttXET16A, AAN87142). Identical amino acids in the sequences are shaded in black. The red box indicates the conserved motif and asteriks (***) mark the N-linked glycosylation motif.

Figure S5. Purification of FvXTH9-His and FvXTH6-His. M, protein marker; CE, crude extract; FT, flow through; A4-, A5-, and A6-fractions were eluted with 250 mM imidazole. Target protein (33 kDa) is shown by arrow.

Figure S6. pH optimum of FvXTH9 (a) and FvXTH6 (b). No XET activity was present in control PYES2. Different buffers were used as follows: sodium acetate buffer (pH 3.6, pH 4.0, pH 4.6, and pH 5.2), sodium succinate buffer (pH 5.0, pH 5.5, pH 6.0, and pH 6.5) and sodium phosphate buffer (pH 6.2, pH 7.0, pH 7.4, and pH 8.0). Each assay was performed as 4 replicates; error bars are standard deviation

Figure S7. Metabolite analysis of *F.* × *ananassa* fruit after agroinfiltration. Relative concentration (‰ equivalent of internal standard of the dry weight) of (a) pelargonidin rutinoside, (b) epiafzelechin pelargonidin glucoside, (c) ellagic acid, (d) kaempferol glucoside, (e) kaempferol glucuronide, (f) kaempferol malonyl glucoside, (g) quercetin glucoside, (h) quercetin glucuronide, (i) catechin, (j) epicatechin dimer, (k) epiafzelechin-epicatechin, (l) p-coumaryl glc ester, (m) ferulic acid glc ester, and (n) caffeic acid glc ester. The data were obtained by analysing 4-6 fruits (10 DPI) for each sample. Control fruit was infiltrated with *A. tumefaciens* AgL0 contained pBI121 empty plasmid. The asterisk indicates statistically significant differences (p < 0.05) between agroinfiltrated fruits with *XTHs* and the empty plasmid.

Figure S8. Amino acid sequence alignment of BdXTH8 (XP_003573584.1), EfHTG (CEH24720.1), FvXTH6 (gene05591), and FvXTH9 (gene01986) as well as pairwise sequence identities using Geneious software with default values.

Table S1. Prediction of the sub-cellular localization of FvXTH9 and FvXTH6.

Table S2. List of primers.
